# Neoadjuvant eribulin mesylate following anthracycline and taxane in triple negative breast cancer: Results from the HOPE study

**DOI:** 10.1371/journal.pone.0220644

**Published:** 2019-08-07

**Authors:** Serena Di Cosimo, Nicla La Verde, Anna Moretti, Marina Elena Cazzaniga, Daniele Generali, Giulia Valeria Bianchi, Luigi Mariani, Valter Torri, Flavio Crippa, Biagio Paolini, Gianfranco Scaperrotta, Maria Carmen De Santis, Massimo Di Nicola, Giovanni Apolone, Alessandro Gulino, Claudio Tripodo, Mario Paolo Colombo, Secondo Folli, Filippo de Braud

**Affiliations:** 1 Fondazione IRCCS Istituto Nazionale dei Tumori—Milano, Italy; 2 ASST Fatebenefratelli-Sacco, PO Sacco, Milano, Italy; 3 ASST Fatebenefratelli-Sacco, PO Fatebenefratelli, Milano, Italy; 4 ASST Monza, Centro di ricerca di Fase 1, Monza, Italy; 5 ASST di Cremona, Cremona, Italy; 6 IRCCS—Istituto di Ricerche Farmacologiche “Mario Negri”, Milano, Italy; 7 University of Palermo, School of Medicine, Palermo, Italy; 8 University of Milan, School of Medicine, Milano, Italy; Istituto di Ricovero e Cura a Carattere Scientifico Centro di Riferimento Oncologico della Basilicata, ITALY

## Abstract

**Background:**

Eribulin mesylate (E) is indicated for metastatic breast cancer patients previously treated with anthracycline and taxane. We argued that E could also benefit patients eligible for neoadjuvant chemotherapy.

**Methods:**

Patients with primary triple negative breast cancer ≥2 cm received doxorubicin 60 mg/m^2^ and paclitaxel 200 mg/m^2^ x 4 cycles (AT) followed by E 1.4 mg/m^2^ x 4 cycles. Primary endpoint was pathological complete response (pCR) rate; secondary and explorative endpoints included clinical/metabolic response rates and safety, and biomarker analysis, respectively. Using a two-stage Simon design, 43 patients were to be included provided that 4 of 13 patients had achieved pCR in the first stage of the study.

**Results:**

In stage I of the study 13 women were enrolled, median age 43 years, tumor size 2–5 cm in 9/13 (69%), positive nodal status in 8/13 (61%). Main grade 3 adverse event was neutropenia (related to AT and E in 4 and 2 cases, respectively). AT followed by E induced clinical complete + partial responses in 11/13 patients (85%), pCR in 3/13 (23%). Median measurements of maximum standardized uptake value (SUVmax) resulted 13, 3, and 1.9 at baseline, after AT and E, respectively. Complete metabolic response (CMR) occurred after AT and after E in 2 and 3 cases, respectively. Notably, 2 of the 5 (40%) patients with CMR achieved pCR at surgery. Immunostaining of paired pre-/post-treatment tumor specimens showed a reduction of β-catenin, CyclinD1, Zeb-1, and c-myc expression, in the absence of N-cadherin modulation. The study was interrupted at stage I due to the lack of the required patients with pCR.

**Conclusions:**

Despite the early study closure, preoperative E following AT showed clinical and biological activity in triple negative breast cancer patients. Furthermore, the modulation of β-catenin pathway core proteins, supposedly outside the domain of epithelial*–*mesenchymal transition, claims for further investigation.

**Trial registration:**

EU Clinical Trial Register, EudraCT number 2012-004956-12.

## Introduction

Triple negative breast cancer (TNBC), which accounts for 10–20% of all breast cancer (BC) cases, is defined by the lack of expression of hormone receptors, estrogen receptor (ER) and progesterone receptor (PgR), and human epidermal growth factor receptor 2 (HER2) [1], and is associated with poor prognosis [2]. Due to the absence of any actionable target, treatment of TNBC relies on chemotherapy since the very early stage of disease. Over the past decades, a large number of adjuvant randomized clinical trials have been conducted to define the optimal chemotherapy, dosing and scheduling, and finally establish the efficacy of anthracycline and taxane-based regimens [3]. Evidence from these trials supports the concept of a threshold effect, thereby indicating that under-dosing of these active agents is associated with a poorer clinical outcome. However, escalation of dosage in dose-dense and dose-intense regimens is still under debate as in general it comes at the expense of increased toxicity [4]. For this reason, research into alternatives to improve the prognosis of TNBC patients has intensified, resulting in the development of new active compounds with a favorable safety profile even after treatment or in combination with anthracycline and taxane [5].

Eribulin mesylate (E, E7389; Halaven) is a non-taxane microtubule dynamics inhibitor able to induce cell cycle arrest in the G2-M phase by forming abnormal mitotic spindles that cannot pass the metaphase/anaphase checkpoint with subsequent apoptosis [6–8].

A pooled analysis of two phase III clinical trials of E in heavily pre-treated metastatic BC patients reported a significant 4.7 month increase in overall survival (OS) compared with controls in the TNBC population [9]. Similarly, the OS in favor of E over capecitabine in Study 301, which involved women with ≤2 prior lines of treatment for advanced disease, was most pronounced in the TNBC population (14.4 vs 9.4 months; p = 0.01) [10]. These observations have sparked considerable interest in the use of E in this BC subtype even beyond the metastatic setting [11–12]. Preoperative systemic (neoadjuvant) treatment of BC yields clinical outcome results similar to those of adjuvant systemic therapy, and improves breast conservation rates because of tumor response to therapy [13]. The preoperative setting also allows monitoring of response to therapy in previously untreated patients. In TNBC, pathological complete response (pCR) at the time of surgery has been shown to correlate with improved disease outcomes in randomized clinical trials suggesting that it may serve as a favorable prognostic factor [14].

Based on the antitumor activity of E in anthracycline and taxane pretreated TNBC patients, and considering the unique opportunity of the neoadjuvant setting as a platform to develop a novel antitumor strategy, we designed the multicenter, prospective, non-randomized, open-label, single-arm, two stage, phase II “Halaven as preOPerative therapy in brEast cancer (HOPE)” trial to evaluate the antitumor activity of E following anthracycline and taxane, and correlative studies attempting to identify predictors of response in localized TNBC patients.

## Materials and methods

### Study patient population

Patients with confirmed clinical stage I-III TNBC and a minimum primary tumor size of 2 cm in largest diameter, assessed by clinical examination and breast imaging (ie mammography ± breast ultrasound ± breast magnetic resonance) were included in the study. Patients with multifocal tumors were eligible if the largest lesion was ≥ 2 cm. Estrogen receptor (ER), progesterone receptor (PgR), and HER2 status were tested locally by immunohistochemistry (IHC). Patients were considered eligible if ER and PgR status was < 1% and HER2 status was 0 or 1+ by IHC; in case of a 2+ result, an in-situ hybridization (FISH/CISH) was performed, and the patient was included when < 4 HER2 copies per nucleus or FISH ratio < 1.8 were found. An Eastern Cooperative Oncology Group (ECOG) performance status (PS) of 0 or 1, adequate bone marrow, renal and hepatic functions, were required. Bilateral or inflammatory breast cancer patients were excluded. Patient recruitment was done consecutively until the desired sample size was reached, and relied on physician referrals in participating sites, including three community hospitals, ie the ASST Monza, Cremona, and Fatebenefratelli-Sacco, and the comprehensive cancer center Fondazione IRCCS Istituto Nazionale dei Tumori—Milano (INT), which coordinated the study.

### Ethical statement

This study was approved by local institutional ethical committees (Comitato Etico ASST Monza, Comitato Etico Val Padana, Cremona, Comitato Etico Milano Area B, Comitato Etico della Fondazione IRCCS Istituto Nazionale dei Tumori—Milano), and was conducted in accordance with the principles of the 18^th^ World Medical Assembly (Helsinki, 1964). All applicable amendments were laid down by the World Medical Assemblies and the ICH guidelines for Good Clinical Practice (GCP). Written informed consent was obtained from each participant in the trial. EUDRACT number was 2012-004956-12.

### Study procedures

In each participating site, patients received doxorubicin 60 mg/m^2^ and paclitaxel 200 mg/m^2^ (AT) on day 1 of a 21-day cycle for a total of 4 cycles [15], followed by E 1.4 mg/m^2^ as a 2–5 min IV bolus injection on days 1 and 8 of a 21 day cycle, for 4 cycles. Complete blood count and biochemistry were required before the initiation of chemotherapy and on day 1 of each cycle with AT, and on days 1 and 8 of each cycle with E. After completion of the four cycles of E, patients underwent surgical excision, carried out 3 to 5 weeks after completion of study treatment. The type of breast surgery and the management of the axilla followed local standard practice. Surgical specimens were collected for pathologic examination and biomarker analysis. The pathological results of the surgical specimens were collected for the purpose of the primary endpoint analysis. After surgery, additional adjuvant chemotherapy ± radiotherapy were permitted. The type of adjuvant treatment was as per investigator´s choice and local standard of care. Patients participated in this study until completion of treatment, unacceptable toxicity, disease progression, or consent withdrawal.

At baseline, tumor assessment included mammography ± breast ultrasound ± breast magnetic resonance imaging according to local expertise (within 21 days of initiating study treatment). Tumor size was measured in centimeters according to RECIST 1.1.

^18^F-FDG PET/CT scan for initial tumor staging, clinical evaluation and standard laboratory tests were required at baseline. Chest ^18^F-FDG PET/CT scan was repeated after 4 cycles of AT, and after 2 cycles of E for the protocol specific purpose of metabolic response evaluation. All ^18^F-FDG PET/CT images had to be taken using the same scanner (hybrid PET/CT system 64 TOF Gemini; Philips Medical Systems) and identical acquisition parameters at INT. Two nuclear medicine reviewers were dedicated to assess both the quality of the scans and the imaging analysis. EORTC criteria were applied to assess tumor response [16], and complete metabolic response was complete resolution of ^18^F-FDG uptake within all lesions, making them indistinguishable from surrounding tissue. Clinical tumor assessment by physical examination was required before the initiation of chemotherapy and on day 1 of each cycle. Tumor radiological re-assessment was done after 3 weeks from the last administration of AT (± 5 days). As per protocol, the end of study visit was scheduled on the day of surgery, though patients were convened for a follow up visit 30 days (with a 7 days window) after definitive surgery. The expected study duration was 18 months for patient accrual, and additional 12 months for safety follow up from the start of study treatment. Data were obtained locally and the central study database was audited by INT.

### Study objectives

The primary objective of this trial was to evaluate the antitumor activity of E combined with an anthracycline/taxane-based regimen given as primary systemic therapy and assessed as pathological complete response rate, defined as the absence of invasive cancer in both the breast and axillary lymph nodes.

Secondary objectives included clinical objective response rate (ORR) as defined by RECIST criteria version 1.1; the overall safety profile and tolerability of E; the association between ^18^F-FDG PET/CT and tumor response in terms of pCR; the predictive value of early ^18^F-FDG PET/CT imaging to evaluate the response to AT, to E and their relationship; as an explorative endpoint, the effect of E following AT on the expression levels of epithelial to mesenchymal transition (EMT) biomarkers.

### Statistical analysis

A Simons’s optimal two-stage design was applied. The sample size was estimated based on expected values of pCR [17] of 40% under the alternative hypothesis and 20% under the null hypothesis. With a type I error of 0.05 and a statistical power of 80%, 13 patients were planned in the first stage. In case of 3 pCRs or fewer in this first stage, the accrual would be stopped. If 4 or more pCRs were observed, accrual would continue to include 43 patients overall. At the end of the trial, if more than 13 pCRs were observed among the 43 patients treated with E following AT the null hypothesis would have been rejected. Estimation and testing of the response rate were based on the exact binomial distribution. Descriptive statistics were used to summarize patient characteristics, diagnosis, treatment administration and compliance, activity endpoints, safety parameters and possibly even cancer biomarkers.

The analyses were conducted using the Statistical Analysis System (SAS, SAS Institute Inc., Cary, NC, USA) and R software.

### Immunohistochemical analysis

Tumor biopsies and surgical tumor tissue samples were fixed in 10% buffered formalin and paraffin embedded. Four-micrometers-thick tissue sections were deparaffinized and rehydrated. The antigen unmasking was performed using Novocastra Epitope Retrieval Solutions pH6; pH 8 and pH 9 in a PT Link pre-treatment module (Dako, Denmark) at 98°C for 30 minutes. The sections were brought to room temperature and washed in PBS. After neutralization of the endogenous peroxidase with 3% H_2_O_2_ and Fc blocking by a specific protein block (Novocastra UK) the samples were incubated overnight with the primary antibodies Rabbit Monoclonal anti-human β-catenin, dilution 1:100 pH8 [Clone 6B3; Cell Signaling]; Rabbit Polyclonal anti-human N-cadherin, dilution 1:500 pH9 [AbCam Code ab18203]; Rabbit Monoclonal anti-human c-myc, dilution 1:500 pH6 [Clone Y69; AbCam Code ab32072]; Mouse Monoclonal anti-human Zeb-1, dilution 1:150 pH6 [Clone 3G6; AbCam Code ab180905]; Mouse Monoclonal anti-human Cyclin D1, dilution 1:25 pH9 [Clone P2D11F11; Leica Novocastra], at 4 C°. Staining was revealed by polymer detection kit (Novocastra) and AEC (3-amino-9-ethylcarbazole) or DAB [3,3- diaminobenzidine tetrahydrochloride] substrate-chromogen. The slides were counterstained with Harris hematoxylin (Novocastra). All the sections were analysed under an AXIO Scope A1 optical microscope (ZEISS) and microphotographs were collected through an Axiocam 503 Color digital camera (Zeiss) using the Zen2 software.

The immunostaining for Cyclin D1, c-myc, and Zeb-1 was scored according to the percentage of positive cells. For the determination of the EMT score, Zeb-1 expression was categorized as follows: 0 negative, 1 around 5%, 2 10–15%, 3 20–25%, 4 30–35%, 5 40–45%, 6 >50%. β-catenin and N-cadherin were scored according to the intensity of the membrane staining as follows: 0 negative, 1 dim expression in less than 50% of cells or intense expression in less than 10% of cells, 2 dim expression in more than 50% of cells or strong expression in >10% <50% of cells, 3 strong expression in more than 50% of cells. The EMT score was calculated as the result of the formula (N-cadherin + Zeb-1 score) ─ β-catenin score. Evaluation of each parameter and EMT scoring were done independently by two pathologists (CT and AG) in a blinded fashion (i.e. without any knowledge of the patient ID, clinical outcome, and order of pre- and post-treatment tumor samples).

## Results

**Fig 1:**
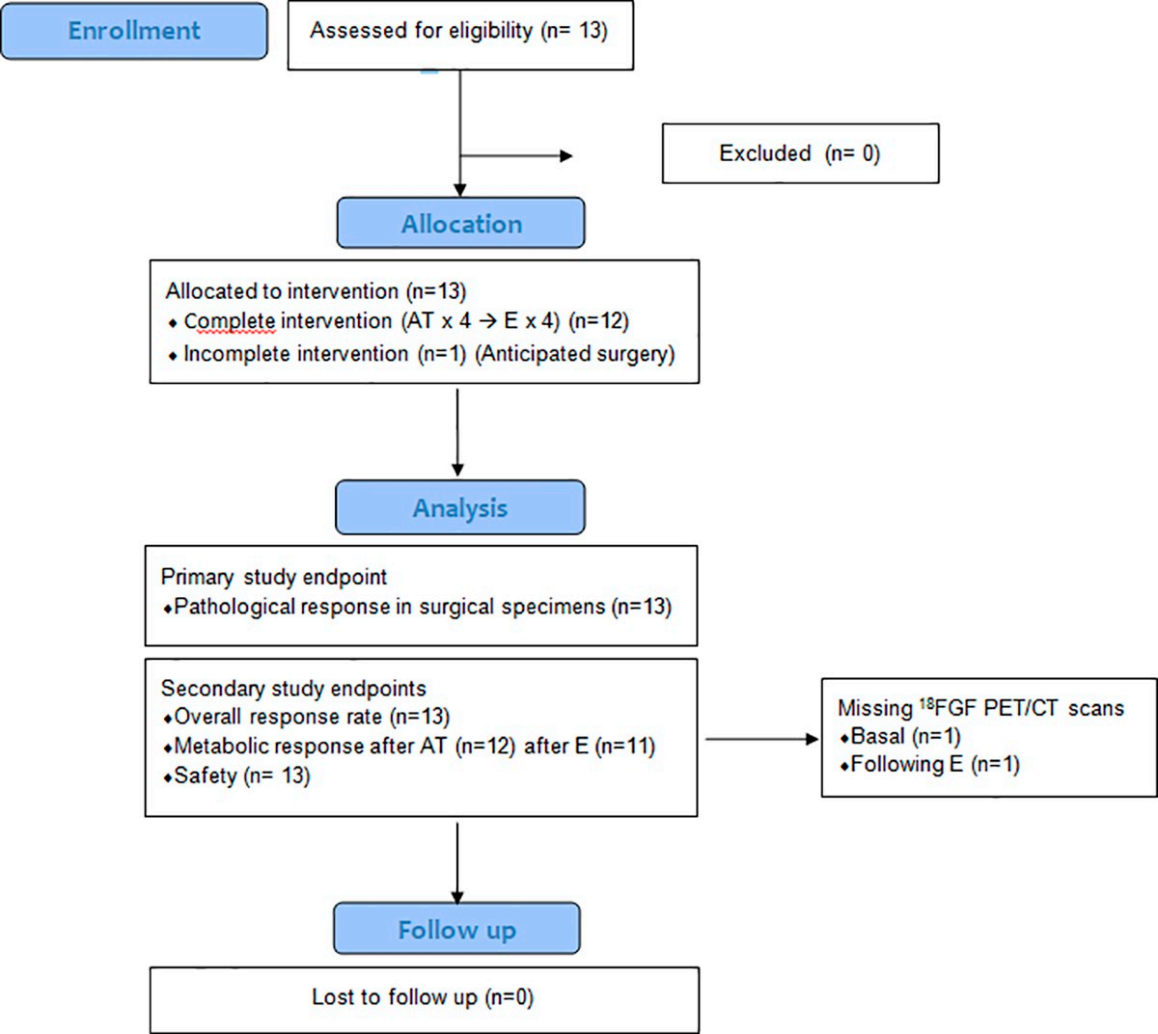
HOPE study flowchart. Doxorubicin/Paclitaxel (AT); Eribulin (E). One patient did not complete the pre planned treatment with AT x 4 cycles followed by E x 4 cycles, as she underwent surgery soon after the second cycle of E, due to progressive disease.

Between August 23, 2013 and March 9, 2015, a total of 13 patients met eligibility criteria and were enrolled in the study. The study flowchart is reported in Fig 1. Patient clinical and pathological features are reported in Table 1. The median age was 43 (range 35–75) years. At diagnosis tumor size was 2–5 cm (cT2) in 9/13 (69%), and > 5 cm (cT3) in 4/13 (30%) patients, with a median value of 4.0 (2.2–7.5) cm; clinical nodal status was positive (cN1-3) in 8/13 (61%) cases. No case of stage I TNBC was enrolled.

**Table 1:** Patients and tumor characteristics.

	N.
**AGE**	
<50 years	9
≥50 years	4
**CLINICAL TUMOR SIZE**	
2–5 cm	9
>5 cm	4
**CLINICAL NODAL STATUS**	
N0	5
N1-3	8
**TUMOR GRADE**	
G2	1
G3	8
Missing	4
**Ki67**	
<50%	4
≥50%	8
Missing	1

Histologic grade 3 was reported in 8/9 (88.8%) evaluable patients. Among 11 evaluable patients, the median Ki67 value was 55% with a range of 25% to 90%. Four and 7 patients had primary tumors with an intermediate (25%–50%) and high (>50%) Ki67 staining, respectively. No case of low Ki67 primary tumor was reported.

Toxicity data were recorded for all 13 patients.

Table 2 lists the most common adverse events before and after treatment with E. Treatment was in general well tolerated. In particular, the most frequent adverse events were represented by G3 and G4 neutropenia, which were related to AT in 4, and to E in 2 cases, respectively.

**Table 2:** Main toxicities.

TOXICITY	ANTHRACYCLINE + TAXANE			ERIBULIN		
	All grades	G3	G4	All grades	G3	G4
**HAEMATOLOGICAL**						
Anaemia	6 (46%)	0 (0%)	0 (0%)	7 (54%)	0 (0%)	0 (0%)
Neutropenia	5 (38%)	1 (8%)	3 (23%)	7 (54%)	2 (15%)	0 (0%)
Leucopenia	1 (8%)	1 (8%)	0 (0%)	2 (15%)	0 (0%)	0 (0%)
**NON-HAEMATOLOGICAL**						
Peripheral neuropathy	6 (46%)	0 (0%)	0 (0%)	7 (54%)	0 (0%)	0 (0%)
ALT	6 (46%)	0 (0%)	0 (0%)	6 (46%)	0 (0%)	0 (0%)
AST	4 (31%)	0 (0%)	0 (0%)	4 (31%)	0 (0%)	0 (0%)
Nausea	4 (31%)	0 (0%)	0 (0%)	4 (31%)	0 (0%)	0 (0%)
Myalgia	4 (31%)	1 (8%)	0 (0%)	4 (31%)	0 (0%)	0 (0%)
Astenia	3 (23%)	0 (0%)	0 (0%)	3 (23%)	0 (0%)	0 (0%)
Vomiting	2 (15%)	0 (0%)	0 (0%)	7 (54%)	1 (8%)	0 (0%)
Mucosal inflammation	2 (15%)	0 (0%)	0 (0%)	3 (23%)	0 (0%)	0 (0%)
Conjunctivitis	1 (8%)	0 (0%)	0 (0%)	6 (46%)	0 (0%)	0 (0%)

Data on objective response rates are summarized in Table 3. Major shrinkage of the primary tumor was measured in 84% of patients (complete response 8%; partial response 76%) after primary AT followed by E. An analysis of the degree of response after AT and after E was feasible in 11 cases. Overall, 6/11 (54%) patients achieved a partial response after AT; 3/5 (60%) patients who did not respond to AT reported a partial response after E; one patient maintained a stable disease, and 1 progressed.

**Table 3:** Clinical and pathological treatment response to E following AT.

**CLINICAL RESPONSE**	**N (%)**
PARTIAL RESPONSE	10 (76%)
COMPLETE RESPONSE	1 (8%)
PROGRESSIVE DISEASE	1(8%)
STABLE DISEASE	1(8%)
**PATHOLOGICAL FINDINGS**	
ypT0N0	3 (23%)
ypT1bN0	3 (23%)
ypT1bN1	1 (8%)
ypT1cN0	3 (23%)
ypT2N0	2 (15%)
ypT2N1	1 (8%)

After primary AT followed by E, 13 patients underwent surgery. Pathological findings were available for all patients. Three out of 13 (23%) patients achieved a less than 1 cm residual disease. Complete absence of invasive cancer in breast and axillary nodes, ie pCR as per protocol definition, was reported in 3/13 (23%) patients. With a median follow up of 49 months (range 47–54), the event free survival (EFS), defined as the time from study treatment initiation to first EFS event (breast cancer relapse, second primary malignancy or death without recurrence) was 61%. None of the patients achieving pCR relapsed.

### Correlative studies

**Fig 2:**
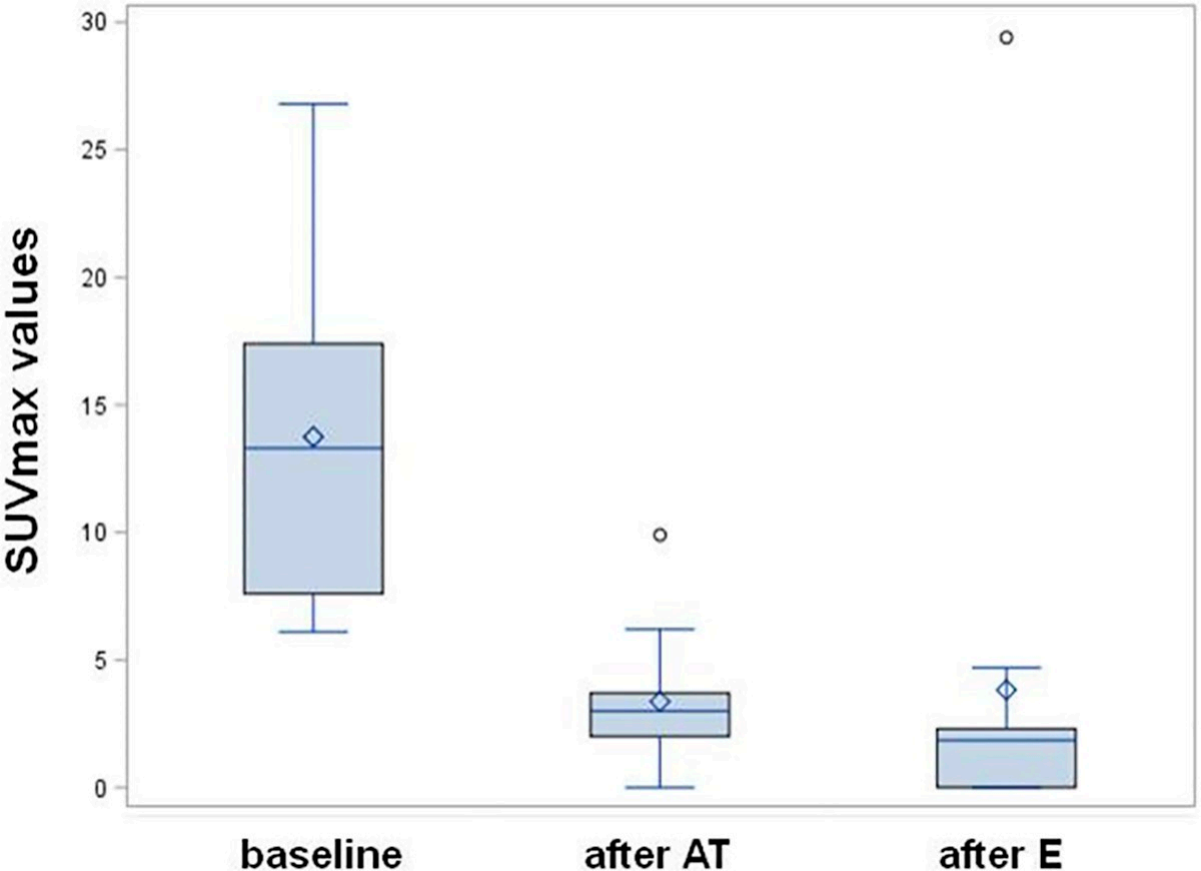
Box- plot of SUV max in TNBC patients at baseline, after treatment with AT, and after E. The boxes represent the 25–75% range with bisecting lines showing the median values, and the horizontal lines represent the 10–90% range.

Chest ^18^F-FDG PET/CT scans were available in 12, 13 and 12 patients at baseline, after AT and after 2 cycles of E, respectively. Measurements of the median maximum Standardized Uptake Value (SUV max) were 13, 3, and 1.9 at baseline, after AT and after E, respectively. As compared to basal values, median SUVmax measurements of primary tumor resulted 26.2% (95%CI 13.2–39.2) and 22.6% (95%CI 0.33–44.9) after AT and E, respectively. Box plots of the changes in SUVmax measurements are shown in Fig 2. Five patients attained a CMR, 2 after AT and additional 3 after E. Two out of these 5 patients achieved pCR at the time of surgery.

**Fig 3:**
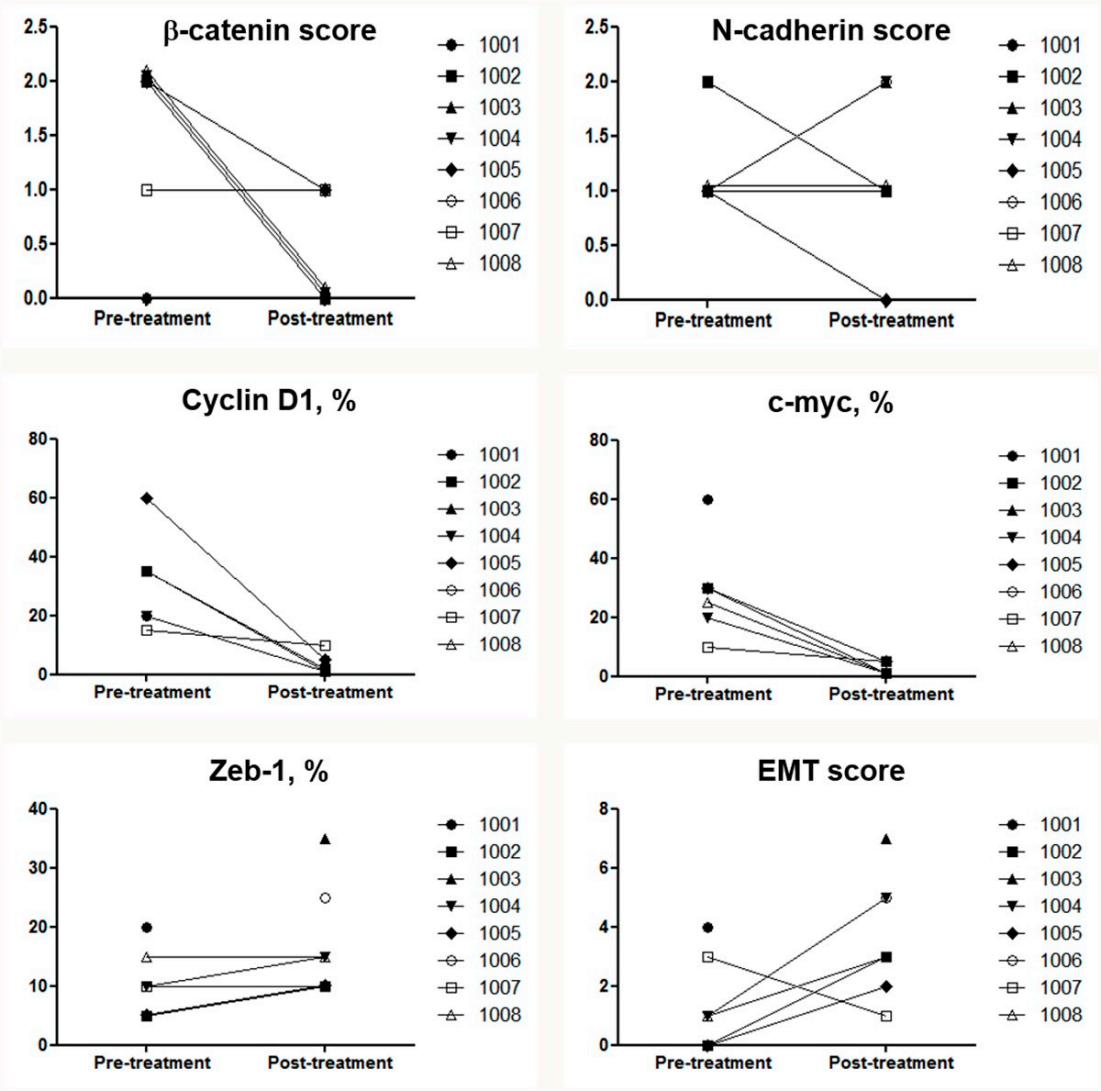
Epithelial to mesenchymal transition biomarker levels in primary tumor samples. Spaghetti plot of the changes of primary tumor immunostaining of individual patients at baseline and after study treatment with neoadjuvant AT followed by E. Immunostaining for the indicated biomarkers was performed on 13 primary tumor specimes, specifically 8 pre-treatment, 5 post-treatment, and 5 paired-biopsies (missing information at surgery in 3 cases: 1 pCR, 2 insufficient amount of residual tumor). Five patients are not represented due to lack of evaluable primary tumor tissue at both baseline and at surgery. Epithelial*–*mesenchymal transition *(*EMT*);* EMT scores, estimated as reported in the Methods section.

Fig 3 shows EMT biomarker levels in all available basal (8 cases) and surgical (5 cases) primary tumor samples. Immunoreactivity for β-catenin and N-cadherin was detected in all primary tumor biopsies at baseline, and resulted moderate in 66% and 50% of cases, respectively. Besides, median pre-treatment rate of positive tumor cells for c-myc, Cyclin D1, and Zeb-1 were 30% (range 10–60%), 50% (15–60%), and 12% (5–25%), respectively. In five cases with paired pre- and post-treatment primary tumor tissue, we observed that β-catenin was undetectable in 3/5 (60%) cases and halved in one additional case after AT followed by E. Notably, this effect was not paralleled by N-cadherin, whose expression fell to zero just in one case. c-myc was undetectable in 60% of cases, Cyclin D1 dropped to levels ≤5% in 80% of cases, whereas the rate of Zeb-1 doubled in 60% of surgical samples (Fig 3).

**Fig 4:**
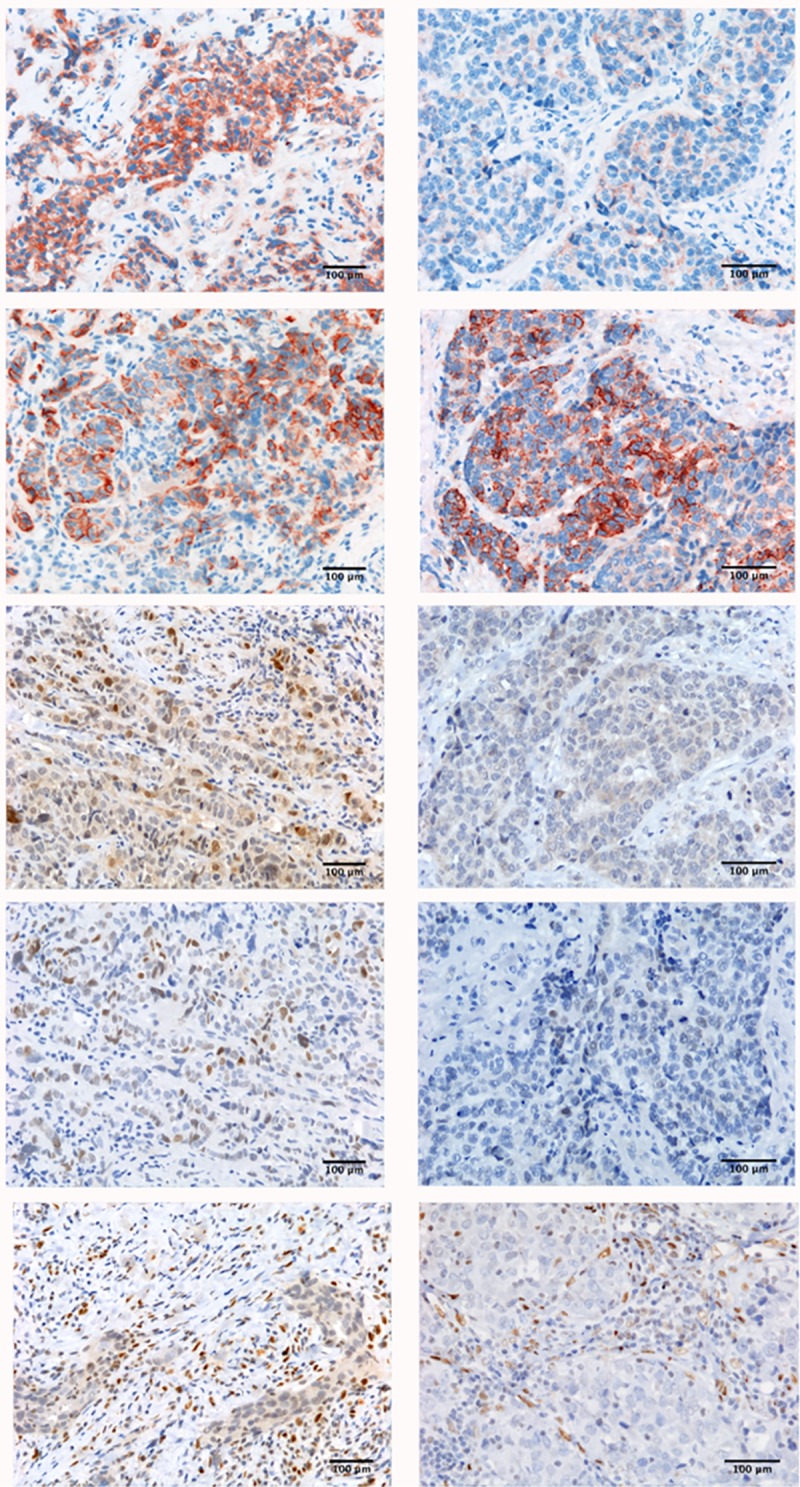
Immune-staining index panel. Specific reaction with β-catenin, N-cadherin, Cylin D1, c-myc and Zeb-1 monoclonal antibodies in primary TNBC before and after neoadjuvant therapy with AT followed by E. Intense immunostaining was evident in pre-treatment primary tumor biopsy, whereas it appeared reduced in surgical specimen after study treatment, with the unique exception of N-cadherin, whose levels remained stable. *Scale bar*, reported at the right bottom of each picture.

Hence, modulation of the Wnt/β-catenin pathway in tumor samples of patients treated with E following AT could involve c-myc signalling, independently of EMT feature changes (immune-staining index panel, showed in Fig 4.

## Discussion

Systemic chemotherapy represents the mainstay of treatment for TNBC and maximizing the rate of pCR is widely considered the most important outcome for preoperative chemotherapy against TNBC. Nevertheless, the optimal neoadjuvant regimen in TNBC patients has not been clearly defined. Current controversies in the field include the role of platinum salts, the optimization of taxane formulations, and the use of biologic agents. Conflicting results of major studies with platinum-based compounds have highlighted the need to balance potential benefits against increased toxicity [18–21]. Data concerning optimal taxane schedules support the use of nab-paclitaxel instead of paclitaxel in selected cases [22–24]. PARP inhibitors deserve additional studies as preliminary data in combination with chemotherapy showed a marginal pCR advantage [25].

The rationale for combination chemotherapy is to use agents with non-overlapping mechanisms of action and established safety profiles. Eribulin mesylate (E) is a new generation chemotherapy agent with a specific mechanism of action and antitumor activity against a wide range of tumor types, including drug-resistant BC. Of note, anthracycline- and taxane-resistant metastatic BC patients are known to be sensitive to E as a single agent [26]. Importantly, significant anticancer activity is seen in ER, PgR and HER2 negative metastatic BC cases [10]. So far few studies evaluating E in localized BC either in combination with carboplatin [27], and trastuzumab [28], or as monotherapy [29] after anthracycline-based chemotherapy have been reported. Overall, results showed that E has a favorable peripheral neuropathy toxicity profile, without any clearly improved activity over historical results with standard treatment with paclitaxel [28]. Our study is the first in literature with neoadjuvant E following anthracycline and taxane in patients with localized TNBC. We aimed to address the antitumor activity of an intense neoadjuvant regimen with doxorubicin and paclitaxel followed by E, and to explore the potential of E anthracycline- and taxane non responders in localized TNBC. Unfortunately, the pCR rate failed to meet our criteria for continuing this trial beyond stage I. Our interim analysis revealed that only three of 13 patients had pCR -below the pre-established futility boundary-so the trial was interrupted.

Several explanations for the lack of E activity in terms of pCR in this trial can be given. We can hypothesize greater activity in patients with earlier-stage disease. Most of the patients enrolled had large primary tumor size and positive nodal status while the study hypothesis was built on prior neoadjuvant studies which also enrolled stage I-IIA breast cancer patients[13–14]. It should be noted that E induced response in 60% of unresponsive cases to doxorubicin and paclitaxel (AT). Albeit the sample size was limited, this “rescue” rate seems to be superior to that reported by Gianni *et al*. with the use of the CMF schema after AT [30]. Furthermore, we showed that E can increase the proportion of patients who achieved a complete metabolic response in TNBC patients who were predicted to be unlikely to respond to AT by ^18^F-FDG PET/CT imaging. Whether this effect will translate into survival benefit remains to be demonstrated in larger study with longer follow up. Although cross-resistance of E with AT can be reasonably excluded, and rather E was developed in patients with anthracycline- and taxane-resistant tumors, the rate of pCR reported in our study is similar to that of anthracycline and taxane-based regimens [13–14]. Nevertheless, E produced a profound down-regulation of EMT biomarkers, as assessed in tumor specimens collected at surgery. Hence, we cannot exclude the possibility of added benefit with E, as it is possible that it may slow progression rather than provide complete tumor response. In fact, both in the EMBRACE study [26], and in the previous phase II studies [31–32], which led to approval of E in the treatment of refractory metastatic BC patients, response rates were in the range of 9% and 12%, yet there was a survival benefit. Furthermore, in a phase II randomized trial evaluating neoadjuvant chemotherapy regimens with weekly paclitaxel or E followed by doxorubicin and cyclophosphamide in women with locally advanced HER2-negative breast cancer, pCR was lower in the E arm than in the paclitaxel one (17 versus 26%) [33]. Therefore, additional research exploring E and more in general EMT targeting drugs is warranted. Indeed, preclinical studies show that E is able to trigger a shift from mesenchymal to epithelial phenotypes via reversal of the EMT to the MET state [34–35]. This transition could be exploitable both to increase drug sensitivity, and to combine with novel compounds, including immunotherapy [36]. Furthermore, a drug capable of reversing EMT could be used to make tumors more sensitive to active drugs on the epithelial component of the tumor. In this sense, E could be explored to be used in sequential schemes to increase sensitivity, rather than to revert the resistance to anthracycline/taxane combinations.

In conclusion, this study failed to demonstrate sufficient E efficacy as measured by pCR. Despite E antitumor activity in anthracycline and taxane resistant BC, it could be possible that E has greater impact upon acquired resistance in metastatic breast cancer. Alternatively, E could have greater disease control rather than cytotoxic effect and pCR could not be the proper endpoint to measure its activity. Interestingly, there are different ongoing studies testing the benefit of E in non-metastatic breast cancer, and we are eagerly awaiting these results (https://www.clinicaltrials.gov). However, since our trial was unable to meet its primary endpoint, we still need to see what ultimate role, and in what clinical context, the modulation of the EMT pathway mediated by E will play in the treatment of TNBC. One possibility is to look at whether reducing EMT correlates with a reshaping of immune microenvironment, which can be in turn exploitable for immune checkpoint Inhibitors combinations.

## Supporting information

S1 TREND Checklist TREND statement checklist 03.01.2019. (DOC)

S1 Study Protocol HOPE clinical study protocol 11.1.2012. (PDF)
