# Supplementary material for: Neoadjuvant eribulin mesylate following anthracycline and taxane in triple negative breast cancer: Results from the HOPE study
Source: PLoS One. 2019 Aug 7;14(8):e0220644. doi: 10.1371/journal.pone.0220644 (PMC6685628; doi:10.1371/journal.pone.0220644)
Supplement: S1 Study Protocol — (PDF) [file pone.0220644.s002.pdf]

CLINICAL STUDY PROTOCOL

STUDY NUMBER: INT 01-13

STUDY NAME: HOPE

Halaven as preOPerative therapy in brEast cancer

COMPOUND: E7389 / ER086526 (Eribulin mesylate)

A phase II, open label, single-arm trial of neoadjuvant therapy in patients with triple negative breast cancer evaluating the efficacy of eribulin mesylate following anthracycline and taxane and correlative science studies attempting to identify predictors of response

PI	Serena Di Cosimo
Document Type	Oncology Clinical Trial Protocol
Version number	00
Development phase	II
Document status	Final
Release date	-November-2012
EUDRACT number	2012-004956-12

Protocol Synopsis

**TITLE**<br><br>**Short title**	A phase II, open label, single-arm trial of neoadjuvant therapy in patients with triple negative breast cancer evaluating the efficacy of eribulin mesylate following anthracycline and taxane and correlative science studies attempting to identify predictors of response.<br><br>**H**alaven as pre**OP**erative therapy in br**E**ast cancer (HOPE study)
**TRIAL LOCATION**	Fondazione IRCCS - Istituto Nazionale dei Tumori di Milano, Italy
**STUDY SPONSOR**	Fondazione IRCCS - Istituto Nazionale dei Tumori di Milano, Italy
**INVESTIGATORS**	Dott.ssa Serena Di Cosimo, Dott.ssa Danila Serpico
**DEVELOPMENT PHASE**	Phase II
**STUDY OBJECTIVE(S)**	**Primary Objectives**<br>• To evaluate the efficacy of the study regimen, assessed by:<br>o Pathological complete response in breast (pCR_B), defined as the absence of invasive tumor cells in the breast<br>o Pathological complete response in breast and axilla (pCR_{BA}), defined as the absence of invasive tumor cells within the breast and axilla<br>**Secondary Objectives**<br>• To estimate the clinical objective response rate (cORR) in the breast and axilla, as defined by RECIST Criteria version 1.1<br>• To assess the breast conservation rate (BCR) at surgery<br>• To determine the overall safety profile and tolerability of the regimen<br>• To estimate the efficacy of eribulin relative to the response to anthracycline/taxane<br>• To establish associations between PET/CT, tumour response and molecular features<br>• To hypothesize about predictive value of early PET/CT imaging for evaluation of response to AT, to eribulin and their relationship
**STUDY RATIONALE**	The neoadjuvant setting provides a unique opportunity to study novel therapeutic agents. Pretreatment biopsies are readily accessible and biomarker/bioimaging results

	can be evaluated against pathologic complete response (pCR), a surrogate endpoint that demonstrates strong association with disease-free and overall patient survival.<br><br>Nevertheless, neoadjuvant therapy with anthracycline and taxane results in pCR in only 10% to 30% of unselected patients with breast cancer. Two studies assessed the role of additional therapy after neoadjuvant anthracycline-based therapy. In the Aberdeen study, patients with large operable or locally advanced breast cancer (LABC) received four cycles of cyclophosphamide/vincristine/doxorubicin/prednisone (CVAP) chemotherapy. Patients with a complete or partial response were then randomly assigned to either four additional cycles of CVAP or four cycles of docetaxel. The addition of sequential docetaxel to neoadjuvant CVAP resulted in an enhanced clinical response rate (94% vs. 66%) and a significantly increased pCR rate (34% vs. 16%; $p < 0.04$) compared with patients receiving CVAP alone *(Heys SD et al. Clin Breast Cancer 3(S2):S69-S74, 2002)*. More recently, the phase III German GEPARTRIO study compared additional docetaxel/doxorubicin/cyclophosphamide (TAC) versus vinorelbine and capecitabine (NX) in patients who did not respond to 2 cycles of neoadjuvant TAC. The rationale for the combination of vinorelbine and capecitabine was to find two active drugs not cross-resistant to standard chemotherapy agents. The pathologic complete response rate was 6.2% for NX, which was not significantly different from the 6.6% pathologic complete response rate seen with additional TAC treatments. Overall, chemotherapy was well tolerated, with NX having a better toxicity profile compared with TAC. These initial TAC non- responders had disappointing pathologic complete response rates, so this trial underscored the point that patients who demonstrate frontline resistance with potent neoadjuvant therapy ultimately do not do well. However, the study demonstrated also that NX was not necessarily successful as salvage therapy for these patients *(Von Minckwitz G et al. J Natl Cancer Inst 2008,100:542-551)*. In summary, available data suggest that there is some benefit, for additional chemotherapy after surgery in these patients unresponsive to neoadjuvant anthracycline with or without taxane-based chemotherapy. This benefit can be further increased as a variety of alternative therapeutic strategies for patients with disease progression on anthracycline/taxane-containing regimens have been explored in recent years.<br><br>E7389 (eribulin mesylate), a structurally simplified

	synthetic analog of halichondrin B, has recently been approved for the treatment of patients with metastatic breast cancer who have received at least two chemotherapeutic regimens for their metastatic disease. In preclinical studies, E7389 induces mitotic arrest and apoptosis in cancer cells, by suppressing microtubule polymerization. Clinical trials evaluating the novel halicondrin analog eribulin, showed the compound to be highly active in anthracycline/taxane refractory advanced breast cancer, including all breast cancer intrinsic sub-type. Among patients with late-line advanced BC previously treated with anthracyclines and taxanes, the response rate with eribulin alone was 12%. More importantly the improvement in response rate was associated with a significant improvement in OS with a HR of 0.81 compared to physician's treatment of choice. Based on these data, sequential treatment with eribulin after anthracyclines- and taxanes-based therapy in the neoadjuvant setting, might offer the dual benefit of ameliorating the response to chemotherapy in AT responsive patients; and more importantly might benefit those patients with unresponsive disease and poor prognosis.<br><br>This will be a phase II, open-label, single-arm, exploratory study of single agent E7389 following anthracycline and taxane as neoadjuvant treatment for stage I-III HER2-negative breast cancer. The study aims to test the efficacy and the safety of the regimen. The ultimate objective of the study is to determine which patients can derive the maximum benefit from treatment with E7389.
**STUDY DESIGN**	Patients will first undergo screening, tumor measurement and collection of core tumor biopsy.<br><br>Following confirmation of eligibility criteria, ER/PgR negative, and type of initially planned surgery (BCS vs. mastectomy) will be recorded.<br><br>After enrollment, the combination of doxorubicin and paclitaxel (AT) will be administered at the doses already published *(Gianni L et al. J Clin Oncology 2009),* every 21 days , for a total of 4 cycles. Then, E7389 will be administered as ready to use solution at 1.23 mg/m^2 (equivalent to 1.4 mg/m^2 eribulin mesylate) as a 2-5 min IV bolus injection on Days 1 and 8 of a 21-day cycle for 4 cycles (Cycles 1-4).<br><br>Clinical tumor measurement by physical examination will be performed and recorded in Day 1 of every cycle.<br><br>Imaging tumor measurement by mammography and ecography/MRI and ecography will be performed at baseline, Cycle 1 Day 1 of E7389 and prior to definitive

	surgery. In such a way, baseline and post- AT treatment tumor assessment should be available for each patient. Relationships between the response to AT and the response to eribulin will be explored. If clinical tumor progression is suspected at any time, an imaging assessment must be obtained for confirmation.<br><br>In order to provide an early and sensitive assessment of the anti-tumor effect of E7389 after treatment with AT in vivo, PET/CT will be performed at baseline, after Cy4 of AT and after Cy2 of E7389.Treatment will be given until definitive surgery, the first sign of disease progression, unacceptable toxicity or withdrawal of patient consent.<br><br>Breast surgery will be carried out 3 to 5 weeks after completion of study treatment. The type of breast surgery and the management of the axilla will follow local standard practices. Surgical specimens will be collected for pathologic examination and biomarker analysis. Patients should return to a follow-up visit 30 days (± 7 days) after definitive surgery. The pathological result of the surgical specimen will be collected for the purpose of the primary endpoint analysis. After surgery, additional adjuvant chemotherapy and adjuvant radiotherapy are permitted. The type of adjuvant treatment will be as per investigator´s choice and local standards of care.
**STUDY POPULATION**<br><br>**Main selection criteria:**	Patients will be eligible for study participation as defined by the following inclusion and exclusion criteria:<br><br>**Inclusion Criteria:**<br><br>• Written informed consent.<br><br>• Age ≥ 18 years.<br><br>• Histologically confirmed and operable invasive breast carcinoma, with all of the following characteristics:<br><br>o Primary tumor ≥ 2cm in largest diameter (cT1-3);<br><br>o cN0-2a;<br><br>o No evidence of distant metastasis (M0).<br><br>• Breast cancer eligible for primary surgery.<br><br>• Available pre-treatment core (Tru-cut) biopsy or possibility of performing one.<br><br>• HER2-negative breast cancer (as per local assessment), defined as either of the following:<br><br>o 0-1+ expression by IHC;<br><br>o 2+ expression by IHC and in situ hybridization (FISH/CISH) without HER2 gene amplification (<4

HER2 gene copies per nucleus, or a FISH ratio [HER2 gene copies to Cr17 signals] of <1.8)

o Is situ hybridization (FISH/CISH) without HER2 gene amplification, independently of IHC.

• ER negative <u>and</u> PgR negative status (as per local assessment).

• In the case of a multifocal tumor (defined as the presence of two or more foci of cancer within the same breast quadrant), the largest lesion must be ≥ 2cm and designated the “target” lesion for all subsequent tumor evaluations and HER2 negative status must be documented in all the tumor foci.

• ECOG performance status of 0 or 1.

• Laboratory values as follows:

o Absolute neutrophil count (ANC) ≥ 1,500/µl.

o Platelets count ≥ 100,000/µl.

o Hemoglobin ≥ 9g/dL.

o Serum bilirubin ≤ 1.5 time the upper limit of normal (ULN).

o Alanine aminotransferase (ALT) and aspartate aminotransferase (AST) ≤ 2.5 x ULN.

o Alkaline phosphatase (AP) ≤ 2.5 x ULN.

o Serum creatinine ≤ 1.5mg/dL or calculated creatinine clearance ≥ 60mL/m.

• Absence of any psychological, familiar, sociological or geographical condition potentially hampering compliance with the study protocol and follow-up schedule.

• Ability and willingness to comply with study visits, treatment, testing, and to comply with the protocol.

Exclusion Criteria:

• Any prior treatment for primary invasive breast cancer.

• Metastatic, locally advanced or inflammatory (i.e. Stage III-IV) breast cancer.

• Bilateral invasive breast cancer.

• Multicentric breast cancer, defined as the presence of two or more foci of cancer in different quadrants of the same breast.

• Pre-existing peripheral neuropathy of any grade.

• Uncontrolled hypertension (systolic >150mmHg and/or diastolic >100mmHg).

• Clinically significant (i.e. active) cardiovascular

	disease.<br>• Concomitant use of inhibitors of hepatic transport proteins such as organic anion-transporting proteins (OATPs), P-glycoprotein (Pgp), multidrug resistant proteins (MRPs) etc. Inhibitors of such transporters include but are not limited to: cyclosporine, ritonavir, saquinavir, lopinavir and certain other protease inhibitors, efavirenz, emtricitabine, verapamil, clarithromycin, quinine, quinidine and disopyramide.<br>• Concomitant treatment with enzyme inducing substances such as rifampicin, carbamazepine, phenytoin, or St John´s wort (Hypericum perforatum). Major medical conditions that might affect study participation (e.g., active peptic ulcer disease, uncontrolled diabetes mellitus, uncontrolled seizure disorder, uncontrolled pulmonary, renal, or hepatic dysfunction, and uncontrolled infection).<br>• Other primary malignant tumors within the previous 5 years, except for adequately controlled limited basal cell carcinoma of the skin or carcinoma in situ of the cervix.<br>• Known human immunodeficiency virus (HIV) infection or other active or serious infection requiring IV antibiotics at randomization.<br>• Pregnancy or breastfeeding women.<br>• Women of childbearing potential (< 2 years after the last menstruation) not using effective, non-hormonal means of contraception (i.e. intra-uterine contraceptive device, barrier method of contraception in conjunction with spermicidal jelly or surgically sterilized) during the study and for a period of 6 months following the last administration of study drug.<br>• Administration of any live virus vaccine within 8 weeks preceding study entry.<br>• Use of any investigational agent within 30 days of administration of the first dose of study drug or concurrent treatment on another clinical study.<br>• Requirement for radiation therapy concurrent with study anticancer treatment.<br>• Known hypersensitivity to any of the study drugs or excipients.<br>Inability or unwillingness to abide by the study protocol or cooperate fully with the investigator or designee.
**TOTAL NUMBER OF PATIENTS:**	Phase II= 43 patients + 5 patients (considering a dropout rate of 10%)
**EXPECTED NUMBER OF SITES:**	Istituto Nazionale dei Tumori di Milano

	Ospedale Fatebenefratelli e Oftalmico di Milano<br>Ospedale San Gerardo di Monza<br>Ospedale Maggiore di Cremona
**STUDY TREATMENTS**<br>**Investigational Product(s)**<br>**Route of administration**<br>**Dose regimen**	• Eribulin mesylate<br>• IV<br>• Eribulin 1.23 mg/m^2 (eribulin mesylate 1.4 mg/m^2) at days 1 and 8 every three weeks<br>For the purposes of scheduling and evaluations, a treatment cycle is defined as 21 days.
**PRIMARY AND SECONDARY ENDPOINT(S)**	**Primary Endpoints**<br>• pCR<br>**Secondary Endpoints**<br>• ORR (complete + partial response)<br>• Metabolic response (complete + partial response)<br>• General safety assessed by monitoring all AEs and serious AEs (SAEs), laboratory measurements, vital signs and physical examination<br>• Percentage of patients with grade 3/4 hematologic toxicity<br>• Dose reductions and dose delays due to treatment toxicity
**ASSESSMENT SCHEDULE**	The study consists of the following procedures:<br><u>Pre-Treatment Period:</u><br>The screening period must occur within 21 days of study treatment initiation (unless noted otherwise) and includes:<br>• Baseline tumor assessment; staging CT scan of the chest/upper abdomen will be performed<br>• Clinical evaluation: complete medical history, menopausal status, physical examination, ECOG status, height, weight, vital signs, and documentation of concomitant medications<br>• Laboratory tests: hematology (with differential, reticulocytes count, and platelets); INR; comprehensive chemistry panel (sodium, potassium, chloride, CO2, creatinine, calcium, BUN, albumin, AST, ALT, alkaline phosphatase, LDH and total bilirubin), serum or urine pregnancy test for women of child bearing potential<br>• LVEF assessment, as per ECHO or MUGA scans

	• 12-lead ECG<br>• ER, PgR, and HER2 status of the tumor<br><u>Study Treatment Period:</u><br>patients may participate in this study until completion of treatment, unacceptable toxicity, disease progression, or consent withdrawal.<br>***Tumor radiological response measurement*** will be performed at baseline, Cycle 1 Day 1 of AT and Cycle 1 Day 1 of E7389 and approximately 21 days after the last drug cycle prior to definitive surgery:<br>Confirmation of clinical response is required<br>***Clinical evaluation*** will be performed prior to each cycle, including but not limited to: physical examination with clinical tumor measurement, ECOG status, vital signs, and documentation of concomitant medications.<br>***Laboratory tests***: hematology (with differential, reticulocytes count, and platelets); INR; comprehensive chemistry panel (sodium, potassium, chloride, HCO3-, creatinine, calcium, BUN, albumin, AST, ALT, alkaline phosphatase, LDH and total bilirubin) on Day 1 of every 3-week cycle before the administration of study treatment.<br>***Adverse events*** will be collected and reported in the CRF.<br><u>Post-Treatment Period:</u><br>Patients will return to the study site within 30 days after the last dose of study treatment for end-of-treatment assessments.
**STATISTICAL CONSIDERATIONS**	A Simon's optimal two-stage design is applied. The sample size is estimated to detect a 40% pCR, with 20% for a minimal hypothesis. With a type I error of 0.05 and a statistical power of 80%, **in the first stage, 13 patients** have to be enrolled. If 4 pCRs or more are observed, accrual will continue to obtain 43 treated patients overall. **At the end of the trial**, if more than 13 pCRs are observed among the **4 patients** treated with AT→ Eribulin the null hypothesis will be rejected. Accrual is planned to stop as soon as the number of required responses by the statistical design are achieved. Estimation and testing of the response rate will be based on the exact binomial distribution.<br>Descriptive statistics will be used to summarize patient characteristics, diagnosis, treatment administration and compliance, activity endpoints, safety parameters and eventually cancer biomarkers. Data will also be displayed graphically, when appropriate.

**DURATION OF STUDY**	18 months from enrollment: The study will comprise patient enrollment and safety follow-up for a duration of one year from the start of study treatment therapy.
**STUDY COMMITTEES**	Steering Committee; Study Management Committee; Safety Independent Committee

Table of Contents

List of Appendices
List of Figures
List of Tables
Glossary of abbreviations
1. Background
  1.1. Triple negative breast cancer
  1.2. Neoadjuvant chemotherapy
  1.3. Neoadjuvant chemotherapy for TNBC
  1.4. ECTO trial
  1.5. Eribulin mesylate (E7389)
    1.5.1. Overview
    1.5.2. Mechanism of action
    1.5.3. Preclinical data
    1.5.4. Clinical data: efficacy and safety
      1.5.4.1. Phase I trials
      1.5.4.2. Phase II trials
      1.5.4.3. Phase III trials
      1.5.4.4. Pharmacokinetic studies
2. Rationale for the study
  2.1. Study rationale and purpose
    2.1.1. Value of taxane in TNBC
    2.1.2. Eribulin in TNBC
  2.2. Rationale for the study design
  2.3. Rationale for dose and regimen selection
3. Objectives and Endpoints
  3.1. Primary objective
  3.2. Secondary objectives
  3.3. Endpoints
    3.3.1. Primary endpoint
    3.3.2. Secondary endpoints
4. Study design
  4.1. Overview
  4.2. Early end of treatment
  4.3. End of study
  4.4. Centers
5. Study population
  5.1. Overview
  5.2. Inclusion criteria
  5.3. Exclusion criteria
  5.4. Concomitant medication and treatment
    5.4.1. Allowed therapies
    5.4.2. Excluded therapies
  5.5. Criteria for premature withdrawal
  5.6. Replacement policy
    5.6.1. For patients
    5.6.2. For centers
6. Study procedures
  6.1. Pre-treatment period
  6.2. Study treatment period
    6.2.1. Day 1 of treatment

6.2.2. Day 1 of cycle 1 of E7389
6.2.3. Day 1 cycle 3 of E7389
6.2.4. Pre surgery visit
6.2.5. Surgery visit
6.3. Proceeding to surgery and safety stopping rules
6.4. After the end of treatment (End of Study Visit)
6.5. Summary table
7. Study committees
7.1. Independent Data Monitoring Committee (IDMC)
8. Investigational Medicinal Products
8.1. Identity and pharmaceutical data of the investigational agents
8.2. Administration of Treatment
8.2.1. Initial dose and treatment plan for AT
8.2.2. Initial dose and treatment plan for E7389
8.2.3. Dose Adjustment for AT
8.2.4. . Dose Adjustments for E7389
8.3. Premedication
8.4. Patient Monitoring
8.5. Duration of therapy
8.6. Concomitant therapy
9. Criteria of evaluation
9.1. Safety evaluation
9.2. Efficacy evaluation
9.3. Pathological evaluation
9.3.1. Definition of Clinical response
9.3.2. Measurability of tumor lesions
9.3.3. Tumor response evaluation
10. Patient safety
10.1. Safety endpoints assessed in this trial
10.2. Adverse Events, Severity and Relationship
10.2.1. Definition of Adverse Event
10.2.2. Assessing Severity of Adverse Events
10.2.3. Assessing Relationship to Study Treatment
10.2.4. Classification of Causality
10.2.5. Pregnancy Reporting
10.3. Serious Adverse Events
10.3.1. Definition of Serious Adverse Events
10.3.2. Reporting of Serious Adverse Events
10.4. Patient temporary or permanent discontinuation and patient study discontinuation
10.4.1. Temporary treatment discontinuation of AT or E7389
10.4.2. Permanent treatment discontinuation of AT or E7389
10.4.3. List of criteria for definitive treatment discontinuation
10.4.4. Handling of patients after permanent treatment discontinuation
10.5. Procedure and consequence for patient withdrawal from study
11. Statistical considerations
11.1. Statistical design and simple size
11.2. Accrual and Duration of Study
11.3. Dataset description
11.4. Analysis Populations
11.5. Baseline characteristics
11.6. Efficacy variables
11.7. Safety Variables

12. Ethical and regulatory standards
    12.1. Ethical principles
    12.2. Laws and regulations
    12.3. Informed consent
    12.4. Institutional Review Board / Independent Ethics Committee
13. Study monitoring
    13.1. Responsibilities of the Investigator(s)
    13.2. Source document requirements
    13.3. Use and completion of Case Report Forms (CRFs) and additional request
    13.4. Use of computerized systems
14. Administrative rules
    14.1. Curriculum Vitae
14.2. Confidentiality
15. Property rights
16. Data protection
17. Insurance compensation
18. PI audits and inspections by regulatory agencies
19. Premature discontinuation of the study or premature close-out of a site
    19.1. Decided by the PI
    19.2. Decided by the Investigator
20. Clinical trial results
21. Publications and communications
22. Clinical trial protocol amendments

List of Appendices

Appendix A: References

Appendix B: ECOG performance status

List of Figures

Figure 1: Chemical Structure of E7389

List of Tables

Table 1. Phase I trials of E7389

Table 2. Phase II trials of E7389

Table 3. Phase III trials of E7389

Table 4. AT dose reductions for the management of toxicity

Table 5. E7389 dose reductions for the management of toxicity

Table 6. Integration of target, non-target and new lesions into response assessment

Table 7. Severity of adverse event

Glossary of Abbreviations

A	Doxorubicin
ABC	Advanced breast cancer
ACT	Doxorubicin and cyclophosphamide followed by docetaxel
AE	Adverse event
ALT	Alanine aminotransferase
ANC	Absolute neutrophil count
AP	Alkaline phosphatase
APTT	Activated partial thromboplastin time
AST	Aspartate aminotransferase
AT	Doxorubicin and paclitaxel
BC	Breast cancer
BCR	Breast conservation rate
BCS	Breast conservative surgery
BSA	Body surface area
BUN	Blood urea nitrogen
CBR	Clinical benefit rate
cm	Centimeter
cORR	Clinical objective response rate
Cr	Cromosom
CR	Complete response
CRF	Case Report Form
CSF	Colony stimulating factors
CT	Computerized Tomography
CTCAE	Common terminology for classification of adverse events
CVAP	Cyclophosphamide/vincristine/doxorubicin/prednisone
DFS	Disease free survival
dL	Deciliter
DLT	Dose limiting toxicity
E7389	Eribulin mesylate
ECG	Electrocardiogram
ECOG	Eastern Cooperative Oncology Group
e-CRF	Electronic clinical report form
EMA	European Medicines Agency
ER	Estrogen receptor

FFPE	Formalin-fixed paraffin embedded
FISH	Fluorescence *in situ* hybridization
G	Tumore grade
GCP	Good Clinical Practice
HER2	Human epidermal growth factor 2
HIV	Human Immunodeficiency Virus
HR	Hazard ratio
IC50	Half maximal inhibitory concentration
ICH	International Conference on Harmonization of Technical Requirements for Registration of Pharmaceuticals for Human Use
IDMC	Independent Data Monitoring Committee
IEC	Independent Ethics Committee
IHC	Immunohistochemistry
INR	International Normalized Ratio
IRB	Institutional Review Board
ITT	Intent-to-treat
IV	Intravenous
Kg	Kilogram
L	Liter
LABC	Locally advanced breast cancer
LDH	Lactate dehydrogenase
LVEF	Left ventricular ejection fraction
m^2	Square meter
MBC	Metastatic Breast Cancer
MedDRA	Medical dictionary for regulatory activities
mg	Milligram
mL	Milliliter
mm	Millimeter
mmHg	Millimeters of mercury
MRI	Magnetic resonance imaging
MRP(s)	Multidrug resistant protein(s)
MTD	Maximum tolerated dose
NAC	Neoadjuvant chemotherapy
NaCl	Sodium chloride
NCI	National Cancer Institute

nM	Nanomolar
NSABP	National Surgical Adjuvant Breast and Bowel Project
NX	Vinorelbine and capecitabine
OATP(s)	Organic anion-transporting protein(s)
ORR	Overall response rate
OS	Overall survival
pCR	Pathological complete response
pCR_B	Pathological complete response in the breast
pCR_{BL}	Pathological complete response in the breast and axillary nodes
PD	Progressive disease
PET/CT	Positron Emission Tomography/Computerized Tomography
PFS	Progression free survival
Pgp	P-glycoprotein
PgR or PR	Progesterone receptor
PK	Pharmacokinetics
PR	Partial response
RD	Residual disease
RECIST	Response Evaluation Criteria In Solid Tumors
RFS	Relapse free survival
RPTD	Recommended phase 2 dose
SAE	Serious adverse event
SC	Steering Committee
SD	Stable disease
SUSAR	Suspected Unexpected Serious Adverse Reaction
T	Paclitaxel
TAC	docetaxel/doxorubicin/cyclophosphamide
TEAE	Treatment emergent adverse event
TNBC	Triple negative breast cancer
TPC	Treatment of physician’s choice
ULN	Upper level of normal
US	Ultrasound
µl	Microliter

1. Background

1.1. Triple Negative Breast Cancer

Breast cancer (BC) is the most frequently diagnosed cancer worldwide and is second only to lung cancer as a cause of cancer death. Until 20 years ago, palliation was the only objective of treatment in advanced BC (ABC): with new effective drugs other goals have been achieved, such as maximum symptom control, prevention of serious complications, and increased survival without diminishing quality of life. As a large proportion of breast cancer cases are now diagnosed in early stages it would be optimal to find a stage-appropriate combination and a correct sequence of surgery, chemotherapy and radiotherapy.

BC is a heterogeneous disease, and not every breast tumor responds equally to a specific agent. Subtypes of BC are distinguished by expression of estrogen receptors (ER), progesterone receptors (PR) and human epidermal growth factor receptor-2 (HER2). Studies based on gene expression profiles have provided additional insights into this complex scenario. Over the past 10 years, four major classes of breast cancer (Luminal A, Luminal B, HER2-enriched, and Basal-like) and a Normal Breast-like group have been identified and intensively studied *(Perou CM, Nature 2000; Sorlie T, Proc Natl Acad Sci USA 2001)*. Known as the "intrinsic subtypes of breast cancer", these groups of tumors have revealed critical differences in incidence, survival and response to treatment. Triple-negative breast cancer (TNBC) is defined by the lack of ER, PR, and HER-2 expression. Preliminary data shows that although TNBC is associated with a poor prognosis, patients with TNBC have increased pathologic complete response (pCR) rates compared with non-TNBC to neoadjuvant chemotherapy (NAC). Recurrence and death rates appear higher for TNBC only in the first 3 years. If pCR is achieved, patients with TNBC and non-TNBC have similar survival. In contrast, patients with residual disease (RD) have worse OS if they have TNBC compared with non-TNBC *(Dent R, Clin Cancer Res 2007; Carey RA, Clin Cancer Res 2007; Liedtke C, J Clin Oncol 2008).*

1.2. Neoadjuvant chemotherapy

NAC, also called primary systemic treatment, is the standard of care for situations in which surgery is contraindicated for example locally advanced, inflammatory or inoperable primary BC *(Bear HD, Semin Oncol 1998; Hortobagyi GN, Cancer 1983; Hortobagyi GN, Cancer 1988; Hortobagyi GN, Cancer 1990)*. Since NAC offers the same survival benefits of adjuvant treatment, it has also moved to the context of operable disease, looking for tumor downstaging and breast conservation surgery *(Fisher B, J Clin Oncol 1998; Wolmark N, NSABBP B-18 J Natl Cancer Inst Monogr 2001)*. Since the response to therapy can be monitored, the patient might also be spared treatment with inactive medications. Patients with pathological complete response (pCR) to NAC have improved disease-free survival (DFS) and overall survival (OS), suggesting its use as a surrogate marker for trials comparing different schedules of primary systemic therapy. So currently, NAC is generally used to improve breast conserving surgery, to assess the response to chemotherapy and to prolong DFS *(Bear HD, NSABBP B-27 J Clin Oncol 2003; Van der Hage JA, EORTC 10902. J Clin Oncol 2001; Kuerer HM, J Clin Oncol 1999).*

Anthracycline-based chemotherapy regimens have been the most extensively studied as primary therapy, but so far no specific regimen has been found to be clearly superior. Regimens tested include combination of cyclophosphamide and 5-fluorouracil with doxorubicin *(Zhang F, Cancer 2003)* or with epirubicin (*Therasse F, J Clin Oncol 2003)*. Since Nineties, taxanes were known to have efficacy in patients with metastatic breast cancer *(Holmes FA, J Natl Cancer Inst 1991; Gianni L, J Natl Cancer Inst 1995; Ravdin PM, J Clin Oncol 1995; Valero V, J Clin Oncol 1995)* but their role was not clear in the adjuvant setting and there was also relatively little data on the comparative efficacy of neoadjuvant and adjuvant regimens. Incorporation of taxanes in neoadjuvant setting has

increased the response rates until 15-20% *(Rastogi P, NSABBP B-18 and B-27. J Clin Oncol 2008; Mazouni C, Ann Oncol 2007; Smith IC, J Clin Oncol 2002; Kaufmann M, J Clin Oncol 2006).* These, however, have not always translated into increased survival. Regimens tested include doxorubicin/cyclophosphamide followed by docetaxel *(Bear HD, J Clin Oncol 2006; von Minckwitz G, GeparDuo trial, J Clin Oncol 2005)*; combination of docetaxel, doxorubicin, cyclophosphamide *(von Minckwitz G, GeparTrio trial, J National Cancer Institute 2008)*; combination of doxorubicin, paclitaxel, cyclophosphamide, methotrexate, fluorouracil *(Gianni L, ECTO trial, J Clin Oncol, 2009)*; addition of capecitabine to a sequence doxorubicin/cyclophosphamide and docetaxel *(von Minckwitz G, GeparQuattro trial, J Clin Oncol 2010)* and a dose-dense sequence of epirubicin and paclitaxel *(Kaufmann M, J Clin Oncol 2006).* Administration strategies have included the sequential, concurrent and both sequential and concurrent delivery of agents as well as dose-dense approaches but so far it is not yet clear which group of patients benefit the most from changing to a non-cross-resistant regimen.

1.3. Neoadjuvant chemotherapy for TNBC

At the present time, chemotherapy is the only proven therapy for TNBC; anthracycline/taxane-based regimens are recommended as neoadjuvant therapy as for non-TN subtypes. However, the dismal prognosis of TNBC patients whose tumors do not achieve a pCR after neoadjuvant chemotherapy with anthracyclines and taxanes renders the development of efficacious new therapies for this BC subtype a major research priority. So, platinum salts are emerging as new effective drugs for TNBC; carboplatin-based schedule shows pCR rates of 54.*6 (Chang HR, Cancer 2010)* against 40% cisplatin-based ones *(Torrisi R, Cancer Chemother Pharmacol 2008).* Cisplatin alone gets a pCR of 20% *(Silver DP, J Clin Oncol 2010)*; importantly, in BRCA1 mutated population, it was reported a RR of 80% with cisplatin monotherapy *(Byrski T, J Clin Oncol 2010).* New schedule for TNBC involve bevacizumab: German group obtains a 39.3% pCR rate by adding bevacizumab to neoadjuvant epirubicin and cyclophosphamide followed by docetaxel *(von Minckwitz G, N Engl J Med 2012)*; similarly the NSABP study shows a 34.5% pCR with the addition of bevacizumab to a docetaxel-based schedule *(Bear HD, N Engl J Med 2012).*

1.4. ECTO trial

The ECTO trial was planned to evaluate the addition of paclitaxel to an anthracycline-based regimen (doxorubicin followed by cyclophosphamide/methotrexate/5-fluorouracil) in adjuvant setting and to compare this combination with the same regimen given as primary systemic therapy *(Gianni L, J Clin Oncol 2009).* The aim was to show that sequential regimen of non-crossresistant cytotoxic drugs, including taxane and doxorubicine, could improve pathologic complete response rate and impact on DFS and OS. So, sequential regimen was chosen as the backbone of therapy. Sequential doxorubicin or epirubicin followed by CMF has shown to be superior to CMF alone *(Buzzoni R, J Clin Onc 1991; Poole CJ, N Engl J Med 2006)* and to alternating doxorubicin and CMF *(Bonadonna G, JAMA 1995; Bonadonna G, J Clin Oncol 2008).* It had also been shown that the duration of adjuvant CMF therapy could be shortened to 6 months *(Tancini C, J Clin Oncol 1983)* and that four cycles of doxorubicin plus cyclophosphamide produced similar results to six cycles of CMF *(Fisher B, J Clin Oncol 1990).*

1355 patients were enrolled in ECTO trial, stratified for T (≤ 4.0 vs >4.0 cm), G (G1 vs G2 vs G3) and HR status (ER/PR positive versus negative). Arm A received doxorubicin followed by cyclophosphamide/methotrexate/5-fluorouracil as postoperative treatment. Arm B and Arm C received doxorubicin/paclitaxel followed by cyclophosphamide/methotrexate/5-fluorouracil as postoperative or preoperative therapy, respectively. After a median follow-up of 31 months, NAC improved RR (CR 49% and PR 29%) and breast-conserving surgery (63% vs 34% of adjuvant therapy) *(Gianni L, Clin Cancer Res 2005; Gianni L, J Clin Oncol 2005*). The most recent analysis was performed after a median 76-months follow-up. Relapse free survival (RFS) improves in Arm B versus Arm A (HR: 0.73). No difference in survival were shown between Arm B and Arm C

(RFS HR 1.21; DRFS HR 1.22; OS HR 1.10). Breast-sparing surgery was 63% in neoadjuvant group versus 34% in Arm B; similarly, pathologic node status was 60% in Arm C versus 39% in Arm B. No difference in local recurrence were reported (4,6% in Arm C versus 4,1% in Arm B). The advantage of adding paclitaxel was seen in all subgroups, including in women with node-negative disease who constituted 40% of patients enrolled in the adjuvant arms. Results from ECTO trial are consistent with literature data of taxane-based schedule superiority to anthracycline-based regimens in terms of recurrence rate, DFS and OS *(De Laurentiis M, J Clin Oncol 2008; Francis P, Natl Cancer Inst 2008)*. Although NAC resulted in a pCR rate of 20% (23% for breast alone) in the ECTO study, this did not translate into a significant improvement in RFS, DRFS or OS compared with the same chemotherapy given postoperatively. These findings are similar to those of the NSABP B27 trial in which addition of docetaxel to AC almost doubled the rate of pCR but this did not cause a significant improvement in DFS and only a marginal improvement in RFS *(Bear HD, J Clin Oncol 2006; Rastogi P, J Clin Oncol 2008).*

1.5. Eribulin mesylate (E7389)

1.5.1. Overview

Halaven® (E7389, eribulin mesylate) is a microtubule inhibitor registered for the treatment of breast cancer after at least two chemotherapeutic regimens for the treatment of metastatic disease. Prior therapy should have included an anthracycline and a taxane in either the adjuvant or metastatic setting. It is a halichondrin B- a natural product originally isolated from the rare marine sponge *Halichondria okadai*-synthetic *(Towle MJ, Cancer Res 2001).*

1.5.2. Mechanism of Action

In preclinical models, E7389 induces mitotic arrest and apoptosis in cancer cells by suppressing microtubule polymerization. Unlike the newer vinca alkaloids, E7389 suppresses centromere dynamics by suppressing relaxation rates and time spent stretching and relaxing, without the corresponding suppressive effects on stretching rates and durations of stretching and relaxation *(Okouneva T, Mol Cancer Ther 2008)*. Furthermore, E7389 sequesters tubulin into non-functional aggregates which compete with soluble tubulin for addition to growing microtubule ends, forming abnormal mitotic spindles that cannot pass the metaphase/anaphase checkpoint *(Jordan MA, Mol Cancer Ther 2005)*. Cell cycle arrests in the G2-M phase with subsequent apoptosis *(Dabydeen DA, Mol Pharmacol 2006; Kuznetsov G, Cancer Res 2004).*

1.5.3. Preclinical Data

In vitro, E7389 inhibits cell growth in MDA-MB-435 (breast cancer), COLO 205 and DLD-1 (colon cancer), HL-60 (promyelocytic leukemia), U937 (histiocytic lymphoma), LNCaP and DU 145 (prostate cancer), and LOX (human melanoma) cell lines with a mean IC_{50} of 1.8 nM (range 0.09-9.5 nM), while IC_{50} values for vinblastine and paclitaxel in the same experiments were 3.2 and 7.3 nM, respectively. E7389 induces G2/M (GAP 2/mitosis stages of cell cycle) cell cycle arrest at 1-10 nM concentrations without affecting progression through the G1 (GAP 1 stage of cell cycle) or S (DNA synthesis stage of cell cycle) cell cycle phases or the G1/S cell cycle transition point *(Towle MJ, Cancer Res 2001)*. The sensitivity of breast cancer cell line cells to E7389 correlates positively with the expression of several beta-tubulin isotypes, including beta-III, beta-V and beta-VI. These data suggest that it may be possible to select patients who, based on patterns of beta-tubulin isotype expression in their tumors, are likely to respond to E7389.

After drug washout mitotic blocks induced by eribulin continue, so its action is irreversible. So, blood levels decrease after drug administration is clinically not significant. In preclinical models, E7389 shows synergistic action with gemcitabine, epirubicin, trastuzumab, cisplatin, docetaxel and

vinorelbine. Combinations of E7389 and docetaxel led to mostly additive inhibition of *in vitro* proliferation of four breast cancer cell lines studied. In *in vivo* experiments, the anticancer effects of E7389 were evaluated in the MDA-MB-435, COLO 205, and LOX xenograft models using 5-6 week-old female Swiss nude mice, and in the OVCAR-3 xenograft model using 7-weekold female BALB/c nude mice. In all four models, treatment with E7389 in the 0.05-1 mg/kg range led to significant anticancer effects, with responses ranging from tumor growth inhibition to tumor regression and eradication of tumors. Additional studies at the United States National Cancer Institute (NCI) confirmed the antitumor activity of E7389 in the MDA-MB-435 xenograft model and demonstrated antitumor activity in the NCI-H522 xenograft model. Intermittent intravenous (IV) treatment with 2.25-9.0 mg/kg E7389 produced 14 of 15 complete tumor regressions in the MDA-MB- 435 model with the average duration of remissions ranging from 24 days (2.25 mg/kg treatment) to 41 days (9.0 mg/kg treatment).

1.5.4. Clinical Data: Efficacy and Safety

1.5.4.1. Phase I Trials

Four mayor phase I dose-finding studies in patients with advanced solid tumors have been completed, using different schedule. In studies dosing on days 1, 8, and 15 of a 28-day cycle, the maximum tolerated dose (MTD) of eribulin mesylate was reported as 1.0 and 1.4 mg/m^2 *(Goel S, Clin Cancer Res 2009; Synold TW, J Clin Oncol 2005)*. Neutropenia was the most commonly reported dose-limiting toxicity (DLT). At the highest dose level tested by Goel et al. (1,4 mg/m^2) 3 patients developed grade 3/4 neutropenia as DLT. In Synold et al. trial, two DLTs were reported at 2.0 mg/m^2, a grade 3 febrile neutropenia and a grade 4 neutropenia. Dosing on day 1 of a 21-day cycle resulted in a MTD of 2.0 mg/m^2; febrile neutropenia developed in all of 3 patients at 4.0 mg/m^2 and 2 of 3 patients at 2.8 mg/m^2 *(Tan AR, Clin Cancer Res 2009).* Dosing on days 1 and 8 of a 21-day cycle led to a maximum tolerated dose of 1.4 mg/m^2 with dose-limiting grade 3 febrile neutropenia in 4 patients and grade 4 neutropenia occurring in 2 of fifteen patients (1 patient in the 1.4 and 2.0 mg/m^2 treatment groups, respectively) *(Minami H, Eur J Cancer Suppl 2008)*. Encouraging tumor response data from these phase I trials led to the initiation of phase II studies in breast cancer.

Table 1. Phase I trials of E7389.

	Schedule	RPTD (mg/m^2)	DLT
**Goel, 2009**	D1, 8, 15 q28d	1	*At 1.4mg/m^2*<br>Neutropenia
**Synold, 2005**	D1, 8, 15 q28d	1.4	*At 2mg/m^2:*<br>Febrile neutropenia<br>Neutropenia
**Tan, 2009**	D1 q21 days	2	*At 4.0 mg/m^2 and 2.8 mg/m^2*<br>Febrile neutropenia
**Minami, 2008**	D1, 8 q21d	1.4	*At 1.4 and 2 mg/m2*<br>Neutropenia<br>Febrile neutropenia

RPTD: Recommended phase II dose. DLT: Dose limiting toxicity.

1.5.4.2. Phase II Trials

Three phase II studies of E7389 in patients with advanced or metastatic breast cancer (MBC) have been completed. All patients had been extensively pretreated and had received a median of three or four prior chemotherapeutic regimens.

Vahdat et al. investigated the efficacy and safety of E7389 in patients with MBC who had received prior treatment with an anthracycline and a taxane (*Vahdat LT, J Clin Oncol 2009*). The median number of previous chemotherapeutic regimens was 4. Based on the results of the Synold phase I study, eribulin mesylate 1.4 mg/m^2 was initially administered as a 2-5 min intravenous infusion on days 1, 8, and 15 of a 28-day cycle. After, the protocol was amended to dosing on days 1 and 8 of a 21-day cycle because of neutropenia occurring in day 15. In 87 patients, E7389 achieved an independently reviewed PR of 11.5% (95% confidence interval 5.7-20.1%) and a clinical benefit rate (considered as PR + stable disease at 6 months) of 17.2% (95% CI 10.0-26.8%). The median duration of response was 5.6 months (range 1.4-11.9), with median PFS of 2.6 months (range 0.03-14.9) and median OS of 9.0 months (range 0.5-27.2), respectively. The most common drug-related grade 3/4 toxicities, as assessed in the safety population (n=103), were neutropenia (64%), leucopenia (18%), fatigue (5%), peripheral neuropathy (5%), and febrile neutropenia (4%).

In a separate study, Cortes et al. investigated the efficacy and safety of E7389 in patients with locally advanced disease or MBC pretreated with anthracycline, taxane, and capecitabine *(Cortes J, J Clin Oncol 2010)*. The median number of previous chemotherapy regimens was 4. Based on Minami phase I trial, patients received eribulin mesylate 1.4 mg/m^2 as a 2-5 min intravenous infusion on days 1 and 8 of a 21-day cycle. In 269 patients, the independently-reviewed ORR was 9.3% (all PR; 95% CI 6.1-13.4%), with a CBR of 17.1%. The median duration of response was 4.2 months, with median reported PFS and OS times of 2.6 months and 10.4 months, respectively. For the safety population (n=291), the most common treatment-related grade 3/4 toxicities were neutropenia (54%), febrile neutropenia (5.5%), leucopenia (14%), and asthenia/fatigue (10%). Grade 3 peripheral neuropathy occurred in 5.5% of patients.

In the Japan study, the safety and efficacy of E7389 was investigated in 81 patients with advanced breast cancer who had previously been treated with an anthracycline and a taxane *(Iwata H, J Clin Oncol 2010)*. This study used Minami schedule, with eribulin mesylate 1.4 mg/m2 as a 2-5 min intravenous infusion on days 1 and 8 of a 21-day cycle. It reported an independently-reviewed ORR of 21.3% (all PR; 95% CI 12.9-31.8). The SD and CBR rates were 37.5% and 27.5% (95% CI 12.9-31.8%), respectively. The median duration of response was 119 days (95% CI 85.0-148.0 days) and the PFS was 112.0 days (95% CI 61.0-133.0 days). OS was 331.0 days (95% CI 234.0-not determined). In line with previous reports, the most common treatment-related grade 3/4 toxicities were neutropenia (95.1%), leucopenia (74.1%), febrile neutropenia (13.6%) and lymphopenia (12.3%). Only 3.7% of patients experienced grade 3 peripheral neuropathy and no grade 4 neuropathic events were reported.

In summary, in all three MBC phase II studies, E7389 had a manageable tolerability profile and the most common drug-related adverse events (AE) were neutropenia, fatigue, alopecia, nausea, and anemia. Additionally, E7389 was associated with a low incidence of peripheral neuropathy limited to grade 3 only. The favorable clinical responses observed in advanced breast cancer supported the undertaking of 2 phase III trials.

Table 2. Phase II trials of E7389.

	**Schedule**	**Number of patients**	**Response**	**Survival**
**Vahdat, 2009**	1,4 mg/m^2<br>D1,8,15 q28d and after D1,8 q21d	87	PR 11.5%<br>CBR 17.2%	PFS 2.6 months<br>OS 9.0 months
**Cortes, 2010**	1,4 mg/m^2<br>D1,8 q21d	269	PR 9.3 %<br>CBR 17.1 %	PFS 2.6 months<br>OS 10.4 months
	1,4 mg/m^2		PR 21.3%	PFS 112.0 days

**Iwata, 2010**	D1,8 q21d	81	CBR 27.5%	OS 331.0 days

PR: partial response; CBR: clinical benefit rate; PFS: progression free survival; OS: overall survival.

1.5.4.3. Phase III Trials

The EMBRACE (Eisai Metastatic Breast Cancer Study Assessing Physician's Choice Versus E7389) trial (NCT00388726) was a phase III study of eribulin mesylate (1.4 mg/m^2 as a 2-5 min intravenous infusion on days 1 and 8 of a 21-day cycle) compared with treatment of physician's choice (TPC) in patients with locally recurrent disease or MBC previously treated with two to five prior chemotherapy regimens, including an anthracycline and a taxane *(Cortes J, EMBRACE trial, Lancet 2011)*. Results from this trial has led to the regulatory approval of eribulin for the treatment of patients with MBC who have previously received at least two chemotherapeutic regimens for the treatment of metastatic disease. Primary endpoint was OS: patients treated with E7389 had a median OS of 13.1 months, compared with 10.7 months for patients in the TPC arm (HR 0.81; 95% CI 0.66-0.99, p=0.04). The median PFS was 3.7 and 2.2 months with E7389 and TPC, respectively (HR 0.87; 95% CI 0.71-1.05; p=0.14). The ORR was 12.2% for patients treated with E7389 and 4.7% for those receiving TPC (p=0.002). Grade 3/4 AEs associated with E7389 were asthenia/fatigue (8.2% grade 3; 0.6% grade 4%), neutropenia (21.1% grade 3; 24.1% grade 4) and peripheral neuropathy (7.8% grade 3; 0.4% grade 4) as observed in previous studies.

A second phase III study (NCT00337103) evaluating the safety and efficacy of E7389 versus capecitabine in extensively pretreated patients with locally advanced disease or MBC has recently completed *(Twelves C, Clin Breast Cancer 2010).*

Table 3. Phase III trials of E7389.

	Study design	Schedule	Survival (months)	Response (%)
**Cortes, 2011**	E7389<br>versus<br>TPC	1,4 mg/m^2<br>D1,8 q21d	PFS: 3.7 vs 2.2<br>OS: 13.1 vs 10.7	ORR 12.2 vs 4.7
**Twelves, 2010**	E7389<br>versus<br>capecitabine	1,4 mg/m^2<br>D1,8 q21d<br>vs<br>2500 mg/m^2/d<br>D1-14 q21d		

TPC: treatment of physician's choice; PFS: progression free survival; OS: overall survival; ORR: overall response rate

1.5.4.4. Pharmacokinetic Studies

Across all clinical trials, E7389 displayed linear pharmacokinetics (PK) over the dose ranges studied. The PK profile was characterized by a rapid distribution phase, extensive volume of distribution, slow to moderate clearance, and a slow elimination. Supporting these findings, a population PK and pharmacodynamic analysis of E7389, developed using pooled data from these and several additional phase I studies was recently reported *(Gupta A, Ann Oncol 2010).*

2. Rationale for the Study

2.1 Study rationale and purpose

2.1.1. Value of taxane in TNBC

Treatment of TNBC relies heavily on different regimes of chemotherapeutic agents but remains one of the most challenging subtypes to treat because of the lack of specific therapies. Despite being

sensitive to chemotherapy, many women with TNBC relapse quickly, developing locoregional recurrence or visceral metastasis. Toxicity and chemotherapy resistance are still major limitations in the treatment of patients with TNBC. Despite current trend of targeted therapy development, cytotoxic agents are a mainstay of treatment of patients with breast cancer. Further research into new cytotoxic compounds is needed in order to maximise benefit, whilst minimising toxicity.

Microtubule inhibitors are among the most frequently used agents for breast cancer chemotherapy, with proven efficacy in both localised and metastatic disease. However, the risk of hypersensitivity reactions and other severe adverse events impairing quality of life, together with susceptibility to resistance, are concerning limitations to their use. Another issue related to this class of agents is peripheral sensory neuropathy, in most of the cases dose-limiting toxicity for patients. Paclitaxel at its MTD produced significant deficits in caudal nerve conduction velocity, caudal amplitude and digital nerve amplitudes, as well as moderate to severe degenerative pathologic changes in dorsal root ganglia and sciatic nerve. The study by the US Oncology Research of Huston and the Sarah Cannon Cancer Center randomized 1830 patients with high risk breast cancer to the standard adjuvant treatment with AC followed by paclitaxel versus the experimental adjuvant treatment with AT followed by paclitaxel (*Loesch DM, ASCO 2007*). At 5-years of follow up, the AT followed by paclitaxel produced significantly better overall survival (p=0.054) and improved DFS (p=0.19). Among TNBC patients both DFS (74% versus 79%, p=0.1) and OS (79% versus 84%, p=0.037) were better in experimental arm. However, the main reasons for patients being taken off study treatment were toxicity (85 patients in the control arm and 128 in the experimental arm) and consent withdrawal (18 patients in the control arm and 30 patients in the experimental arm). For this reason, research into alternatives has intensified, thus resulting in the discovery and development of new compounds with a more tolerable profile as compared with paclitaxel.

2.1.2. Eribulin in TNBC

Halichondrins were the most recent class of tubulin inhibitors to enter clinical development, and eribulin has recently been European Medicines Agency (EMA) approved for advanced or metastatic breast cancer in patients who have progressed after at least two chemotherapeutic regimens for advanced disease and who received prior anthracycline and taxane regimens where suitable. Approval was based on the results of the global phase III EMBRACE study assessing treatment of the physician's choice versus eribulin, which demonstrated a statistically significant increase in OS compared with TPC (median OS 13 versus 11 months, HR 0.81; $P = 0.041$) (*Cortes L, Lancet 2011*). Among the total of 762 patients, 19% had TNBC. Of note, eribulin was most effective in hormone receptor-negative patients who had a 34% decreased risk of death compared with TPC chemotherapy, and in TNBC patients, who had a 29% risk reduction. Treatment with eribulin was well tolerated (*Pean E, Clin Cancer Res 2012*). Neutropenia, leucopenia, peripheral neuropathy, and asthenia/fatigue were the most common adverse events reported at Common Terminology Criteria for Adverse Events (CTCAE) grades 3 and 4. Neutropenia was the most common adverse events reported at CTCAE grade 4 in the eribulin group (24.1%). Based on findings to date, eribulin is an attractive agent, and its role in earlier settings of the disease, as well as potentially in combination with antracycline and taxane, deserves further investigation.

2.2 Rationale for the study design

This study is a phase II, open label, single-arm trial of neoadjuvant therapy in patients with triple negative breast cancer evaluating the efficacy of eribulin mesylate following anthracycline and taxane and correlative science studies attempting to identify predictors of response.

Eligible patients will receive AT every 3 weeks for 4 cycles followed by eribulin mesylate 1.4 mg/m^2 every 3 weeks for 4 cycles.

Drugs are given in sequence according to the evidence provided in the literature. Indeed, the sequential administration of chemotherapy has been reported to be more effective than concurrent administration in women with operable node-positive breast cancer. The sequential ACT regimen — doxorubicin and cyclophosphamide followed by docetaxel — had a greater impact on disease-free survival than a doxorubicin/docetaxel combination or concurrent ACT. According to this study, sequential administration of chemotherapy provided a significant 17% reduction in mortality, compared with concomitant regimen (p = 0.03). It also showed a non significant 14% reduction in mortality compared with concurrent regimen (p =0 .09). Disease-free survival was also better with sequential treatment than with the 2 other therapeutic regimens

In order to provide an early and sensitive assessment of the anti-tumor effect of E7389 after treatment with AT *in vivo,* PET/CT will be performed at baseline, after Cy4 of AT and after Cy2 of E7389. The value of PET/CT as a tool for monitoring patients response has been recently reviewed (*Cochet A, J Natl Cancer Inst 2011*).

2.3 Rationale for dose and regimen selection

As previously reported (Sections 1.4 and 2.1.1), the combination of AT has been widely investigated for the treated of breast cancer patients both in the neo- and adjuvant setting. Herein, patients will be treated with doxorubicin 60 mg/m^2 and paclitaxel 200 mg/m^2 every 3 weeks for 4 cycles. Thereafter, eribulin mesylate will be given at the dose of 1.4 mg/m^2 days 1,8 every 3 weeks for additional 4 cycles.

3. Objectives and endpoints

3.1. Primary Objective

The primary objective of this trial is to evaluate the efficacy of addition of eribulin to an anthracycline/taxane-based regimen given as primary systemic therapy and assessed as pathologic complete response rate, both in breast and axilla nodes. The aim is to testify that sequential regimen of non-crossresistant cytotoxic drugs, including taxane and doxorubicine, could improve pathologic complete response rate as still established in ECTO trial *(Gianni L, J Clin Oncol 2009).*

3.2. Secondary Objectives

This study has the following secondary objectives:

- To estimate the clinical objective response rate (cORR) in the breast and axilla, as defined by RECIST Criteria version 1.1.
- To assess the breast conservation rate (BCR) at surgery
- To determine the overall safety profile and tolerability of eribulin given after 4 cycles of doxorubicin plus paclitaxel
- To estimate the efficacy of eribulin relative to the response to anthracycline/taxane
- To establish associations between PET/CT, tumour response and molecular features
- To hypothesize about predictive value of early PET/CT imaging for evaluation of response to AT, to eribulin and their relationship.

3.3. Endpoints

3.3.1. Primary Endpoint

- Pathologic complete response rate in breast (pCR_B), defined as the absence of invasive tumor cells in the breast and pathological complete response in breast and axilla (pCR_{BA}), defined as the absence of invasive tumor cells within breast and axilla.

3.3.2. Secondary Endpoints

- Clinical and radiological ORR, defined by RECIST 1.1 as complete response plus pathologic partial response rate. Physical examination, breast mammography and/or breast ultrasound (US) and/or breast magnetic resonance imaging (MRI) will be used to evaluate clinical and radiological response
- Rate of methabolic response defined a reduction of SUV at PET/CT scan and evaluate as complete or partial response
- Percentage of patients with grade 3/4 hematologic toxicity
- Dose reductions and dose delays due to treatment toxicity
- Safety and adverse events, assessed by the NCI Common Terminology for Classification of Adverse Events (CTCAE) v.4, including the following parameters:
    - General safety, assessed by monitoring all AE and serious AE (SAE), laboratory measurements, vital signs, and physical examination.
    - Percentage of patients with neutropenia Grade 3/4.
    - Percentage of subjects with neuropathy.
    - Incidence of myalgia/arthralgia.

4. Study Design

4.1. Overview

This is a prospective, non-randomized, open-label, multicenter, single-arm study of doxorubicin/paclitaxel followed by eribulin mesylate as neoadjuvant therapy in patients with Stage I-III triple negative breast cancer.

The primary endpoint of the trial is the pathologic complete response rate in both breast and axillary nodes.

A signed written informed consent form must be obtained before any study specific assessment is initiated. However, procedures conducted as part of the subject's routine clinical management (e.g. diagnostic tumor biopsy, or diagnostic imaging studies) obtained prior to signing of informed consent may be utilized for screening or baseline purposes provided these procedures are conducted as specified in the protocol and within the protocol-defined timeframes.

Patients will first undergo screening, tumor measurement and collection of core tumor biopsy if not previously done. Following confirmation of eligibility criteria, patients will be enrolled. Ki67 (%) and type of initially planned surgery (BCS vs. mastectomy) will be recorded. In the case of a multifocal tumor, the "target" lesion will be clearly identified.

After enrollment, doxorubicin will be administered at 60 mg/m^2 and paclitaxel at 200 mg/m^2 on day 1 of a 21-days cycle for a total of 4 cycles as already published *(Gianni L, J Clin Oncol 2009)*. After 3 weeks, eribulin mesylate will be administered at 1.4 mg/m^2 as a 2-5 min IV bolus injection on days 1 and 8 of a 21-days cycle, for a total of 4 cycles.

Clinical tumor measurement by physical examination will be performed and recorded in day 1 of every cycle of treatment.

Tumor radiological response measurement will be performed at baseline, cycle 1 day 1 of AT and cycle 1 day 1 of E7389 and prior to definitive surgery. All these measurements must be recorded.

If clinical progression of the breast tumor is suspected at any time, an imaging assessment (mammography and breast US or breast MRI and breast US) must be obtained for confirmation. In the presence of discordant clinical and imaging evaluations, decision on the maintenance of study treatment will be made by agreement between the investigator and the medical coordinator of the study, and always in the best interest of the patient. If progressive disease (PD) is confirmed, the

treatment with AT or E7389 will be withheld, and further treatment will be delivered according to investigator's decision.

Breast surgery will be carried out 3 to 5 weeks after completion of study treatment. The type of breast surgery and the management of the axilla will follow local standard practices. Surgery specimens will be collected for histologic examination.

Baseline and post-treatment (surgical) primary breast tumor tissue samples should be available for each patient for molecular characterization. A minimum of 2 core formalin–fixed paraffin–embedded (FFPE) and fresh-frozen tissues will be collected at each time point. Biopsies should not be performed in pre-irradiated fields. In case of multifocality, samples will always be collected from the lesion that was defined as "target lesion" at the time of the enrollment.

The end of study visit will be performed at the day of surgery. Patients should return to a follow-up visit 30 days (with a 7 days window) after definitive surgery. The pathological result of the surgical specimen will be collected for the purpose of the primary endpoint analysis. Toxicities related to the study treatment will be followed-up by the investigator at least every 4 weeks after the last visit, until toxicity related to the study treatment resolves, stabilizes, returns to basal level or is considered irremediably irreversible.

After surgery, adjuvant radiotherapy is recommended.

4.2. Early End of Treatment

If clinically evident PD, unacceptable toxicity or withdrawal of patient consent occurs at any time, neoadjuvant administration of AT or E7389 will be stopped prior to the completion of the planned four plus four cycles of treatment.

If treatment is stopped for any of these reasons, further treatment will be delivered according to investigator's decision. Both changing to a different chemotherapy regimen and proceed to definitive surgery are acceptable options.

For patients who stop treatment for progressive disease or unacceptable toxicity further treatment will be as per investigator's decision.

4.3. End of Study

The end of study for primary analysis will be the date of last patient having definitive surgery.

4.4. Centers

This is a multicenter trial involving 4

5. Study Population

5.1. Overview

A total of 43+5 patients with triple negative invasive breast cancer will be enrolled in this study. Patients will be eligible for study participation as defined by the following inclusion and exclusion criteria:

5.2. Inclusion Criteria

<u>All</u> of the following criteria must be fulfilled for a patient to be eligible to this study:

1) Written informed consent must be given according to ICH/GCP guidelines and national/local regulations before patient registration.

2) Age ≥ 18 years.
3) Histologically confirmed and operable invasive breast carcinoma, with all of the following characteristics:
    a) Primary tumor ≥ 2cm in largest diameter (cT1-3) assessed by physical examination and mammography and/or breast US and/or breast MRI (where available). If the tumor is not palpable or measures less than 2 cm in physical examination, the patient is eligible if the imaging technique (mammography and/or breast US and/or breast MRI) shows a tumor of at least 2 cm.
    b) cN0-2a: patients with clinically evident axillary lymph nodes can be included, but not patients with clinically involved internal mammary or supraclavicular nodes (i.e., cN2b-3).
    c) No evidence of distant metastasis (M0).
4) Breast cancer must be eligible for definitive primary surgery.
5) Available pre-treatment core (Tru-cut) biopsy or possibility of performing one.
6) HER2-negative breast cancer (as per local assessment), defined as either of the following:
    a) 0-1+ expression by IHC;
    b) 2+ expression by IHC and in situ hybridization (FISH/CISH) without HER2 gene amplification (<4 HER2 gene copies per nucleus, or a FISH ratio [HER2 gene copies to Cr17 signals] of <1.8)
    c) Is situ hybridization (FISH/CISH) without HER2 gene amplification, independently of IHC.
7) ER negative and PgR negative status (as per local assessment).
8) Known percentage of Ki67-positive tumor cells (as per local assessment).
9) In the case of a multifocal tumor (defined as the presence of two or more foci of cancer within the same breast quadrant), the largest lesion must be ≥ 2cm and designated the “target” lesion for all subsequent tumor evaluations and HER2 negative status must be documented in all the tumor foci. ER/PgR status of the “target lesion” will be considered to classify the breast cancer subtype, as determined by IHC criteria.
10) ECOG performance status of 0 or 1.
11) Laboratory values as follows:
    a) Absolute neutrophil count (ANC) ≥ 1,500/µl.
    b) Platelets count ≥ 100,000/µl.
    c) Hemoglobin ≥ 9g/dL.
    d) Serum bilirubin ≤ 1.5 time the upper limit of normal (ULN).
    e) Alanine aminotransferase (ALT) and aspartate aminotransferase (AST) ≤ 2.5 x ULN.
    f) Alkaline phosphatase (AP) ≤ 2.5 x ULN.
    g) Serum creatinine ≤ 1.5mg/dL or calculated creatinine clearance ≥ 60mL/m.
12) Absence of any psychological, familial, sociological or geographical condition potentially hampering compliance with the study protocol and follow-up schedule.
13) Ability and willingness to comply with study visits, treatment, testing, and to comply with the protocol; those conditions should be discussed with the patient before registration in the trial.

5.3. Exclusion Criteria:

A subject will not be eligible for inclusion in this study if <u>any</u> of the following criteria apply:

1) Any prior treatment for primary invasive breast cancer.
2) Metastatic, locally advanced or inflammatory (i.e. Stage III-IV) breast cancer.
3) Bilateral invasive breast cancer.

4) Multicentric breast cancer, defined as the presence of two or more foci of cancer in different quadrants of the same breast.
5) Pre-existing peripheral neuropathy of any grade.
6) Uncontrolled hypertension (systolic >150mmHg and/or diastolic >100mmHg).
7) Clinically significant (i.e. active) cardiovascular disease, including congenital long QT syndrome, cerebrovascular accident (≤ 6 months before enrolment), myocardial infarction (≤ 6 months before enrolment), unstable angina, New York Heart Association ≥ grade 2 congestive heart failure, serious cardiac arrhythmia requiring medication during the study and that might interfere with regularity of the study treatment, or not controlled by medication.
8) Concomitant use of inhibitors of hepatic transport proteins such as organic anion-transporting proteins (OATPs), P-glycoprotein (Pgp), multidrug resistant proteins (MRPs) etc. Inhibitors of such transporters include but are not limited to: cyclosporine, ritonavir, saquinavir, lopinavir and certain other protease inhibitors, efavirenz, emtricitabine, verapamil, clarithromycin, quinine, quinidine and disopyramide.
9) Concomitant treatment with enzyme inducing substances such as rifampicin, carbamazepine, phenytoin, or St John´s wort (Hypericum perforatum).
10) Major medical conditions that might affect study participation (e.g., active peptic ulcer disease, uncontrolled diabetes mellitus, uncontrolled seizure disorder, uncontrolled pulmonary, renal, or hepatic dysfunction, and uncontrolled infection).
11) Other primary malignant tumors within the previous 5 years, except for adequately controlled limited basal cell carcinoma of the skin or carcinoma in situ of the cervix.
12) Known human immunodeficiency virus (HIV) infection or other active or serious infection requiring IV antibiotics at randomization.
13) Pregnancy or breastfeeding women.
14) Women of childbearing potential (< 2 years after the last menstruation) not using effective, non-hormonal means of contraception (i.e. intra-uterine contraceptive device, barrier method of contraception in conjunction with spermicidal jelly or surgically sterilized) during the study and for a period of 6 months following the last administration of study drug.
15) Administration of any live virus vaccine within 8 weeks preceding study entry.
16) Use of any investigational agent within 30 days of administration of the first dose of study drug or concurrent treatment on another clinical study.
17) Requirement for radiation therapy concurrent with study anticancer treatment. Patients who require breast or chest wall radiation therapy after surgery are eligible.
18) Known hypersensitivity to any of the study drugs or excipients.
19) Inability or unwillingness to abide by the study protocol or cooperate fully with the investigator or designee.

5.4. Concomitant Medication and Treatment

5.4.1. Allowed Therapies

Concomitant treatments are any prescription medications, over-the-counter preparations, herbal remedies or radiotherapy used by a patient in the interval beginning 7 days prior to the patient being recruited into the study and continuing through the study. All concomitant medications should be reported to the Investigator and recorded on the case report form (CRF). In general, all medications taken by the patient for concomitant diseases should continue during the study treatment period and should be recorded on the eCRF.

The following treatments are permitted during the study:

- Acceptable methods of contraception must be used when the female patient or male partner is not surgical sterilized or does not meet the study definition of postmenopausal 12 months of amenorrhea).
- H1 and H2 antagonist (e.g. diphenhydramine, cimetidine)
- Analgesics (e.g. paracetamol/acetaminophen, meperidine, opioids)
- Short term use of corticosteroids to treat or prevent allergic or infusion reactions
- Antiemetics (approved prophylactic serotonin-antagonists, benzodiazepines, ondansetron etc)
- Medication to treat diarrhea (e.g. loperamide)

5.4.2. Excluded Therapies

The following therapies are excluded during the treatment period of the study:

- Anti-cancer therapies other than those administered in this study, including cytotoxic chemotherapy, radiotherapy, (except for adjuvant radiotherapy for breast cancer after completion of chemotherapy or additional adjuvant chemotherapy immediately postsurgery, if deemed necessary) immunotherapy, and biological anticancer therapy.
- Colony stimulating factors (e.g. G-CSF)
- Any targeted therapy
- Treatment with steroids except for thyroid hormone replacement therapy and short term corticosteroid, in order to treat or prevent allergic or infusion reactions.
- High doses of systemic corticosteroids. High dose is considered as > 20 mg of dexamethasone a day (or equivalent) for > 7 consecutive days.
- Any investigational agent, except for those used for this study.
- Initiation of herbal remedies. Herbal remedies initiated prior to study entry and continuing during the study are permitted and must be reported on the appropriate CRF.
- Any oral, injected or implanted hormonal methods of contraception.

5.5. Criteria for Premature Withdrawal

Patients have the right to withdraw from the study at any time for any reason. The Investigator also has the right to withdraw patients from the study in the event of intercurrent illness, adverse events, and treatment failure after a prescribed procedure, protocol violation, administrative reasons or for other reasons. An excessive rate of withdrawals can render the study uninterpretable; therefore, unnecessary withdrawal of patients should be avoided.

In the case that the patient decides to prematurely discontinue study treatment ("refuse treatment"), she should be asked if she can still be contacted for further information. The outcome of that discussion should be documented in both the medical records and in the CRF.

5.6. Replacement Policy

5.6.1. For Patients

Patients enrolled into the study will be replaced until a withdrawn of 10%. We consider to enroll up to 5 further patients.

5.6.2. For Centers

A center may be replaced for the following administrative reasons:
– Excessively slow recruitment.
– Poor protocol adherence.

6. Study Procedures

During the course of the study, all enrolled patients must be evaluated according to the schedule outlined in the flowchart and described below.

6.1. Pre-treatment period

The informed consent will have to be signed by the patient before any procedure specific to the study is performed. However, procedures conducted as part of the subject's routine clinical management (e.g. diagnostic tumor biopsy, or diagnostic imaging studies) obtained prior to signing of informed consent may be utilized for screening or baseline purposes provided these procedures are conducted as specified in the protocol and within the protocol-defined timeframes.

The following assessments and procedures must be performed within 7 days of initiating study treatment (unless noted otherwise):

- Clinical evaluation, including:
  - Demographics, with date of birth;
  - Menopausal status;
  - Complete medical history, including details of the malignancy (date of diagnosis, primary tumor histology, grade, Ki67 expression and stage) and any history of previous or present neuropathies;
  - Current and concomitant medications;
  - Complete physical examination, with special attention to breast tumor measurement (caliper preferred) and axilla examination;
  - ECOG performance status;
  - Height (in centimeters) and weight (in kilograms);
  - Vital signs (blood pressure, pulse rate and temperature);
  - Baseline signs and symptoms, according to the NCI CTCAE v.4.0.
- Baseline mammography plus breast US or breast MRI plus US (within 21 days of initiating study treatment). Tumor size will be measured in cm, and according to RECIST 1.1. The same technique selected at baseline must be consistently used throughout the trial for tumor assessment.
- Chest radiography and abdominal US, to rule out metastatic disease. A bone scan should be obtained only if bone metastases are clinically suspected.
- PET/CT scan for metabolic evaluation of the lesion.
- Laboratory tests:
  - Hematology tests, with differential and platelets count;
  - Coagulation panel, including INR and APTT;
  - Comprehensive chemistry panel: sodium, potassium, chloride, creatinine, calcium, blood urea nitrogen (BUN), albumin, AST, ALT, AP, LDH and total bilirubin;
  - Serum pregnancy test for women of child bearing potential. This test must be obtained within 15 days of beginning study treatment.
- 12-lead ECG and echocardiogram with LVEF measurement.
- ER, PgR, and HER2 status in the target breast lesion. Ki67 (%) will also be collected.
- Baseline tumor tissue collection for molecular-biological tests. A minimum of 2 core FFPE and 2 fresh-frozen tissue samples will be obtained.
- Documentation of the type of initially planned breast surgery (BCS vs. mastectomy).

After confirming all eligibility criteria, patients will be registered and assigned a study number.

6.2. Study treatment period

Patients will participate in the study until planned surgery after 4 cycles of study treatment, or until unacceptable toxicity, PD or consent withdrawal occurs.

6.2.1. Day 1 of treatment

In Day 1 of each cycle, the following assessments will be performed:

- Complete physical examination, with special attention to breast tumor measurement (caliper preferred) and axillary status.
- ECOG performance status.
- Vital signs.
- Body weight.
- Collection of any AEs and SAEs, with assignment of the appropriate adverse event grade according to NCI CTCAE v.4.0.
- Documentation of concomitant medications.
- Laboratory tests: hematology tests (with differential, reticulocytes, and platelets count); coagulation panel (including INR and APTT) and comprehensive chemistry panel (sodium, potassium, chloride, CO_2, creatinine, calcium, BUN, albumin, AST, ALT, AP, LDH and total bilirubin).

6.2.2. Day 1 cycle 1 of E7389

The pre-eribulin visit will be performed before administration of the first dose of E7389. The Following assessments will be performed:

- Complete physical examination, with special attention to breast tumor measurement and axillary status.
- ECOG performance status.
- Vital signs.
- Body weight.
- Collection of any AEs and SAEs, with assignment of the appropriate adverse event grade according to NCI CTCAE v.4.0.
- Documentation of concomitant medications.
- Laboratory tests: hematology tests (with differential, reticulocytes, and platelets count); coagulation panel (including INR and APTT) and comprehensive chemistry panel (sodium, potassium, , creatinine, calcium, BUN, albumin, AST, ALT, AP, LDH and total bilirubin).
- Tumor radiological assessment. This assessment should be done 3 weeks after the last administration of AT (± 5 days). Mammography plus breast US or breast MRI plus US will be performed, accordingly to the method that was used at baseline to define the tumor size of ≥ 2 cm.
- ECG and echocardiogram with LVEF measurement.

6.2.3. Day 1 cycle 3 of E7389

This visit will be performed before administration of the third dose of E7389. The following assessments will be performed:

- Complete physical examination, with special attention to breast tumor measurement and axillary status.
- ECOG performance status.

- Vital signs.
- Body weight.
- Collection of any AEs and SAEs, with assignment of the appropriate adverse event grade according to NCI CTCAE v.4.0.
- Documentation of concomitant medications.
- Laboratory tests: hematology tests (with differential, reticulocytes, and platelets count); coagulation panel (including INR and APTT) and comprehensive chemistry panel (sodium, potassium, , creatinine, calcium, BUN, albumin, AST, ALT, AP, LDH and total bilirubin).
- PET/CT scan for methabolic response evaluation.

6.2.4. Pre-surgery visit

The pre-surgery visit will be performed 3 weeks (± 1 week) after the administration of the last dose of E7389, and always within one week prior to the definitive surgery. The visit may be in the same day as surgery.

The following assessments will be performed:

- Complete physical examination, with special attention to breast tumor measurement (caliper preferred) and axillary status.
- ECOG performance status.
- Vital signs.
- Body weight.
- Collection of any AEs and SAEs, with assignment of the appropriate adverse event grade according to NCI CTCAE v.4.0.
- Documentation of concomitant medications.
- Laboratory tests: hematology tests (with differential, reticulocytes, and platelets count); coagulation panel (including INR and APTT) and comprehensive chemistry panel (sodium, potassium, , creatinine, calcium, BUN, albumin, AST, ALT, AP, LDH and total bilirubin).
- Tumor radiological assessment. This assessment should be done 3 weeks after the last administration of E7389 (± 5 days). Mammography plus breast US or breast MRI plus US will be performed, accordingly to the method that was used at baseline to define the tumor size of $\geq$ 2 cm.
- PET/CT scan for methabolic response evaluation.
- ECG and echocardiogram with LVEF measurement.

6.2.5. Surgery visit

This visit occurs in the day of the surgery. The following procedures will be done:

- Collection of tumor tissue from the surgical specimen for molecular analysis.
- Immediately after surgery: recording of the type of breast surgery performed (BCS vs. mastectomy).

6.3. Proceeding to surgery and safety stopping rules

Experimental treatment will be delivered until:

- Completion of 4 cycles of treatment *or*
- Patient's consent withdrawal *or*
- Progressive disease *or*
- Unacceptable toxicity.

Patients who complete all 8 cycles of study treatment will undergo surgery according to local practices. A minimum of 3 weeks and a maximum of 5 weeks are required between the last study

treatment administration and surgery, unless surgery has to be postponed beyond 5 weeks for toxicity reasons.

Patients who withdraw consent will go off protocol, and no further procedures in the study will be performed neither will they be considered for the primary endpoint analysis.

If clinically PD occurs at any time, a mammography plus a breast US or a MRI plus US must be obtained to confirm progression. If PD is not confirmed by imaging techniques, the patient may continue the study treatment as planned.

If clinical and/or radiological PD is observed at any time, experimental treatment will be withheld and further treatment will be administered at the discretion of the investigator.

Patients who don't complete the planned 8 cycles of treatment for intolerable toxicity or disease progression can proceed directly to surgery or receive other type of neoadjuvant treatment, at the discretion of the investigator and in the best interest of the patient.

If a rate of PD >15% is observed after the first 20 patients are included, then the trial will be permanently stopped

An interim analysis will be performed after the first 25 patients are enrolled and decisions on treatment continuation will be made.

6.4. After the end of treatment (End of Study Visit)

Patients will return to the study site 30 days (± 7 days) after surgery for end-of-treatment assessments, as follows.

- Complete physical examination.
- ECOG performance status.
- Vital signs.
- Body weight.
- Collection of any AEs and SAEs, with assignment of the appropriate adverse event grade according to NCI CTCAE v.4.0.
- Documentation of concomitant medications.
- Laboratory tests: hematology tests (with differential, reticulocytes, and platelets count); coagulation panel (including INR and APTT) and comprehensive chemistry panel (sodium, potassium, , creatinine, calcium, BUN, albumin, AST, ALT, AP, LDH and total bilirubin).
- Assessment of the presence of tumor tissue in the surgical specimen collected at the time of definitive surgery.

Following surgery, patients will be treated as per local standards of care at the discretion of the investigator. It is recommended that patients should receive radiotherapy as per local practices.

6.5. Summary table

	Screening	Cycle 1 AT Day 1	Cycle 2/3/4 AT Day 1	Cycle 1 E7389 Day 1	Cycle 2/3/4 E7389 Day 1	Pre-Surgery	End of study
**Informed consent [a]**	X						
**Inclusion / exclusion criteria**	X						
**Clinical history [b]**	X						
**ER / PgR / HER2 assessment**	X						
**Pregnancy test (if applicable) [c]**	X						
**Evaluation of AE [d]**	X	X	X	X	X	X	X
**Concomitant medication**	X	X	X	X	X	X	X
**Complete physical examination [e]**	X	X		X		X	
**Brief physical examination**			X		X		X
**Breast palpation, tumor measurement and axillary status [f]**	X	X	X	X	X	X	X
**ECOG status**	X	X	X	X	X	X	X
**Vital signs [g]**	X	X	X	X	X	X	X
**Laboratory tests [h]**	X	X	X	X	X	X	X
**Tumor radiological assessment [i]**	X			X		X	
**Staging assessments [j]**	X			X		X	
**Methabolic evaluation[k]**	X		X (only after Cy4)		X (only after Cy2)		
**Administration of the study treatment**		X	X	X	X		
**ECG**	X			X		X	
**Echocardiogram (LVEF %)**	X			X		X	
**Type of surgery: planned**	X						

**Type of surgery: performed**							X

[a] Written informed consent must be obtained prior to any study specific procedure.

[b] Including demographics, menopausal status, details of the malignancy (date of diagnosis, primary tumor histology, grade and stage) and any history of previous or present neuropathies.

[c] Serum pregnancy test for women of child bearing potential. This test must be obtained within 15 days of beginning study treatment.

[d] According to the NCI CTCAE v.4.0.

[e] Including weight (in kilograms) and height (in centimeters; only in the screening visit)

[f] Caliper measurement preferred for tumor size assessment.

[g] Blood pressure, pulse rate and temperature.

[h] Hematology (differential, reticulocyte, and platelets count), coagulation panel (including INR and APTT) and comprehensive chemistry panel (sodium, potassium, chloride, CO2, creatinine, calcium, BUN, albumin, AST, ALT, AP, LDH and total bilirubin).

[i] Mammography and breast US or MRI and US. Tumor size will be measured according to RECIST 1.1. The same technique selected at baseline must be consistently used throughout the trial for tumor assessment.

[j] Chest radiography and abdominal US. Bone scan only if bone metastases are suspected.

[k]PET/CTscan.

7. Study Committees

7.1 Independent Data Monitoring Committee (IDMC)

The IDMC will convene approximately every six months – either face-to-face or via teleconference – to review accrual, study conduct (including treatment withdrawals), patient safety, and disease-related events (with particular attention to premature termination of the study treatment due to disease progression). The IDMC will report its recommendations to the protocol study chair.

8. Investigational Medicinal Products

8.1. Identity and pharmaceutical data of the investigational agent

Name: Eribulin mesylate.

Development Code: E7389

Laboratory Code: ER-086526

NIH/NCI No.: NSC-707389

Chemical Name (Chemical Abstract Services): 11,15:18,21:24,28 – Triepoxy -7,9 – ethano - 12,15 – methano - 9*H*,15*H*furo [3,2.i] furo [2',3':5,6] pyrano [4,3-b] [1,4] dioxacyclopentacosin - 5(4*H*)-one, 2 - [(2*S*) - 3-amino - 2-hydroxypropyl] hexacosahydro - 3-methoxy - 26-methyl - 20,27-bis (methylene) -, (2*R*, 3*R*, 3a*S*, 7*R*, 8a*S*, 9*S*, 10a*R*, 11*S*, 12*R*, 13a*R*, 13b*S*, 15*S*, 18*S*, 21*S*, 24*S*, 26*R*, 28*R*, 29a*S*) -, methanesulfonate salt.

Chemical Formula: C40H59NO11·CH3SO3H

Molecular Weight: 826.00

Chemical Structure:

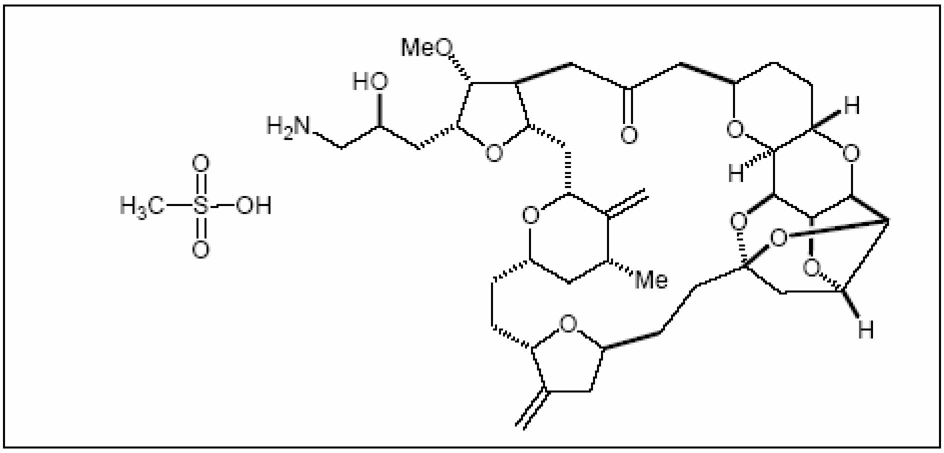

Figure 1: Chemical Structure of E7389.

Appearance: White powder

Hygroscopicity: E7389 is hygroscopic.

Solubility Profile: The solubility of E7389 was determined at room temperature in various organic solvents and water. E7389 was at least freely soluble in *N*-methyl-2-pyrrolidone, methanol, ethanol, acetonitrile, and ethyl acetate; soluble in acetone and 1-octanol; sparingly soluble in water; and very slightly soluble in methyl-*tert*-butylmethyl ether and heptane. E7389 in aqueous buffers (Britton-Robinson buffer, ionic strength = 0.3, pH 2 to pH 11, 25°C) was at least sparingly soluble.

Drug Product: E7389 Eisai Standard 1.0 mg (as mesylate salt) in 0.1 mL ethanol plus 1.9 mL Water for Injection.

8.2. . Administration of Treatment

Before dose administration, the amount of drug needed for each patient must be calculated in the following manner:

$$\text{Scheduled dose (mg/m}^2\text{) x body surface area (BSA) (m}^2\text{) = Dose (mg)}$$

BSA will be calculated using any method that is accepted and customarily used by the clinical site, such as the Mosteller formula:

$$\text{BSA (m}^2\text{) = ([Height (cm) x Weight (kg)]/3600)½}$$

Height and body weight will be recorded at the Screening visit. Thereafter, body weight will be recorded on Day 1 of each treatment cycle for calculation or adjustment of BSA, and consequent adjustment of dose. However, the dose will not be adjusted for body weight on Day 8 of the treatment cycles.

8.2. 1. Initial dose and treatment plan for AT

Patients will receive a slightly lower dose of doxorubicin (60 mg/m^2) immediately followed by paclitaxel 200 mg/m^{2}infused over 3 hours. After premedication (see Section 8.5) patients will receive doxorubicin diluted in 40 mL of normal saline infused in no more than 15 minuts. After 15 minutes they will start paclitaxel diluted in 500 mL of D5W as closely as possible to the beginning of the infusion. Treatment will be administered on day 1 every 21 days for a total of 4 cycles or until PD, unacceptable toxicity or withdrawal of patient's consent. If holidays or personal schedules make administration on day 1 impossible, then administration may be 1 day earlier or 2 days later. In case of longer delay for reasons other than toxicity, this must be discussed and approved by the Sponsor. Regardless of the time of administration, toxicity from previous treatment must be within acceptable ranges as described below.

8.2. 2. Initial dose and treatment plan for E7389

Each vial contains 1 mg of E7389 in 2 mL solution (0.5 mg/mL).

Dose (mg) x 2 = the number of mL of E7389 to withdraw from vials for administration.

The amount of E7389 required (calculated above) will be withdrawn from the appropriate number of vials into a syringe. This may be injected directly as an IV bolus over 2-5 minutes or diluted in up to 100 ml 0.9% NaCl for IV infusion over 2-5 minutes.

Therapy will be administered on days 1 and 8 every 21 days, for a total of 4 cycles or until PD, unacceptable toxicity or withdrawal of patient's consent.

Treatment will be administered on days 1 and 8 whenever possible. If holidays or personal schedules make administration on days 1 or 8 impossible, then administration may be 1 day earlier or 2 days later. In case of longer delay for reasons other than toxicity, this must be discussed and approved by the Sponsor. Regardless of the time of administration, toxicity from previous treatment must be within acceptable ranges as described below.

8.2.3. Dose Adjustment for AT

Doses will be reduced for haematological and other adverse events. Dose adjustments are to be made according to the greatest degree of toxicity. Adverse events will be graded using the NCI CTCAE Version 4. The guidelines which follow outline dose adjustments for toxic effects. If a patient experiences several adverse events and there are conflicting recommendations, please use the recommended dose adjustment that reduces the dose to the lowest level.

The major adverse effects of AT which limit dose are neutropenia / febrile neutropenia and neurotoxicity. Toxicities will be managed by treatment interruptions and dose reductions. Once the dose has been reduced, it cannot be increased at a later date, even if previous toxicity is completely resolved

Table 4. AT dose reductions for the management of toxicity.

	**Reccomended dose**
**ANC < 1500 x 10^9/L or**<br>**PLT < 100 x 10^9/L**	Delay **AT** 1 week
**ANC ≥ 1000 but < 1500 x 10^9/L or**<br>**PLT ≥ 100 but < 150 x 10^9/L**<br>**after delay 1 week**	**A** 50 mg/m^2<br>**T** 150 mg/m^2
**ANC < 1000 x 10^9/L or**<br>**PLT < 100 x 10^9/L**<br>**after delay 1 week**	Delay 1 more week before using doses as above
**ANC < 500 x 10^9/L or**<br>**PLT < 50 x 10^9/L**	**A** 50 mg/m^2<br>**T** 150 mg/m^2

Non-hematological toxicity

- Gastrointestinal: in case of stomatitis or diarrhea G2 delay 1 week or till G ≤ 1; if G > 2 reduce **doxorubicin** dose to 50 mg/m^2 after resolution to G ≤ 1;
- Neurological: if neuropathy G > 2 reduce **paclitaxel** to 150 mg/m^2;
- Cardiac: discontinued **doxorubicin** in case of congestive heart failure, persistent arrhythmia, decrease of LVEF < 45%;
- Hypersensitivity: discontinued **paclitaxel** infusion and administer
    1. chlorphenamine 10 mg IV,
    2. IV fluids, if hypotension
    3. adrenaline SC every 15-20 minuts, if wheezing
    4. nebulized albuterol solution 0.35 cc, if no response to adrenaline
    5. methylprednisolone 125 mg IV to prevent recurrent or late reactions.

If hypersensitivity is G > 2, the patient should not receive paclitaxel again.

If hypersensitivity is G ≤ 2, the patient should be pre-treated with:

1. dexamethasone 8 mg per dose IV 24, 18, 12, 6 hours before paclitaxel;
2. chlorphenamine 10 mg IV 30 minuts before paclitaxel;
3. cimetidine 300 mg IV 30 minuts before paclitaxel;
4. paclitaxel diluted in 1000 mL.

8.2.4. Dose Adjustments for E7389

Doses will be reduced for haematological and other adverse events. Dose adjustments are to be made according to the greatest degree of toxicity. Adverse events will be graded using the NCI CTCAE Version 4. The guidelines which follow outline dose adjustments for toxic effects. If a patient experiences several adverse events and there are conflicting recommendations, use the recommended dose adjustment that reduces the dose to the lowest level.

The major adverse effects of E7389 which limit dose are neutropenia / febrile neutropenia and neurotoxicity. Toxicities will be managed by treatment interruptions and dose reductions. Once the dose has been reduced, it cannot be increased at a later date, even if previous toxicity is completely resolved.

Treatment in subsequent cycles

Treatment for a subsequent cycle will only occur if all of the following verify at day 1 of the Cycle:

- ANC ≥ 1.0 x 10^9/L;
- Platelets ≥ 75 x 10^9/L;
- All other toxicity of a previous cycle has recovered to ≤ Grade 2 (except anemia, alopecia or inadequately treated nausea or vomiting) or to Grade ≤ 1 in the prensence of Grade 3 neurotoxicity.

If treatment has to be postponed for any of these reasons, the first day of treatment will be considered day 1 of the next cycle. If treatment is delayed for 2 weeks or more as a result of toxicity, the patient should be withdrawn from the study, unless discussed and agreed with the Sponsor.

Dose modifications on Day 1

Please refer to **Table 5** for dose level reductions of E7389.

Dose level of E7389 *will be reduced* at day 1 in the case of:

- Hematological grade 3-4 toxicities (as described below) in the previous cycle, recovered to grade ≤ 2:
    - Grade 4 neutropenia (ANC < 0.5 x 10^9/L) lasting more than 7 days in the previous cycle;
    - Grade 3 or 4 neutropenia (ANC < 1 x 10^9/L), in the presence of fever or infection requiring treatment with growth factors and/or antibiotics;
    - Grade 4 thrombocytopenia (platelets < 25 x 10^9/L);
    - Grade 3 thrombocytopenia (platelets < 50 x 10^9/L), in the presence of bleeding and/or requiring blood or platelet transfusion;
- Non-hematological grade 3 or 4 toxicities in the previous cycle, recovered to grade ≤ 2 within 7 days, with or without maximal supportive care (except alopecia);
- Omission of Day 8 administration in previous cycle for toxicity
- Note: If grade 3-4 hematologic toxicities do not recover to ≤ grade 2 in two weeks, or non-hematologic grade 3-4 toxicities to ≤ grade 2 in one week, the patient should be removed from the study; however, if the patient is deemed to have clinical benefit from treatment**,** continuation of treatment and reduction of the dose must be discussed with the sponsor
- Dose modifications on Day 8
- If the conditions to continue treatment, as specified in Section 8.4.4 do not verify at Day 8 of the cycle, treatment on that day will be postponed until recovery to those values.

If recovery occurs on or before Day 15, treatment will be resumed at that time. The new day of treatment administration will be considered the **new Day 8.** The dose will then be reduced one level.

If toxicity has not recovered to the above values on Day 15, the second administration in this cycle will be omitted and treatment will resume as scheduled on Day 1 of the next cycle.

Dose reductions for E7389

Table 5 shows the recommended dose reductions for E7389.

Table 5. E7389 dose reductions for the management of toxicity.

	**Recommended dose**
**First event**	1.1 mg/m^2
**Second event**	0.7 mg/m^2
**Third event**	Consider discontinuation

8.3.. Premedication

Corticosteroids and antihistamines premedication is required before AT administration. All patients must receive:

- Prednisone 25 mg p.o. the evening before therapy
- Hydrocortisone 250 mg IV 30 minuts before paclitaxel
- Chlorphenamine 10 mg IV or i.m. 30 minuts before paclitaxel
- Cimetidine 300 mg IV 30 minuts before paclitaxel

Alternative drugs may be used if those are not commercially available.

No specific premedication is required prior to the administration of E7389.

8.4. Patient Monitoring

Some patients may experience asymptomatic bradycardia during paclitaxel infusion. Hypersensitivity reactions are possible and usually occur within the first hour from the beginning of the infusion in the first and, less frequently, second cycle. It is recommended that patient's blood pressure is monitored during infusion and that patient is supervised for one more hour after the end of paclitaxel administration.

No specific patient monitoring is required during or after the infusion of E7389.

8.5. Duration of Therapy

Treatment will be given for 4+4 cycles, unless PD is observed (see definition in Section 9.3.3) or unacceptable toxicity is present.

Patients may also discontinue protocol therapy in the following instances:

- Intercurrent illness which would in the judgment of the investigator affect patient safety, the ability to deliver treatment or the primary study endpoints;
- Patient withdrawal of consent.

8.6. Concomitant therapy

All diagnostic, therapeutic, or surgical procedures relating to malignancy should be recorded in the CRF including the date, indication, description of the procedure(s) and any clinical findings.

All concomitant treatment or medication administered during the 30 days preceding first administration of the investigational product and throughout the study until 30 days after the final administration of the investigational product, must be reported on the CRF. The generic name of the drug (or trade name for combination drugs) must be specified along with the total daily dosage, route, duration of treatment, and indication of use.

Any medication considered necessary for the patient's welfare that is not expected to interfere with the evaluation of the study drug may be given at the discretion of the Investigator. Ancillary treatments will be given as medically indicated.

Any changes in documented, permitted concomitant treatment already being taken at the beginning of the clinical study must be recorded in the space in the CRF reserved for this purpose, noting the type of medication, the dose, duration, and indication.

Permitted concomitant treatments:

- Anti-emetics.
- Anti-diarrhea therapy.
- Analgesics.
- Anti-allergic measures such as corticosteroids and antihistamines.
- Patients who are being treated with bisphosphonates when they enter the study may continue the medication as long as the dose is stable. If a change in dose is deemed necessary, the case must be discussed with the Sponsor. If a patient requires initiation of bisphosphonates after starting study treatment, this may be permitted on discussion with the Sponsor.
- A number of medications are permitted with caution. Drugs that are weak CYP3A4 inhibitors include saquinavir, nefazodone, fluconazole, grapefruit juice, fluoxetine, fluvoxamine, zileuton and clotrimazole. The investigator should monitor patients carefully for potential adverse interaction between these medications and E7389. In case of suspicion of adverse interactions, these medications should be discontinued with careful follow up of the patient until such interaction is resolved. A complete list of drugs that are substrates, inducers or inhibitors of CYP3A4 can be found at http://medicine.iupui.edu/flockhart.

Concomitant treatment that are not permitted:

- The use of primary prophylaxis with white blood cell growth factors is not allowed. However, following an episode of febrile neutropenia or a delay in treatment administration due to neutropenia, the use of white blood cell growth factors is permitted as judged clinically appropriate. The use of erytropoetin is allowed.
- Other anti-cancer or investigational agents.
- Inhibitors of hepatic transport proteins such as organic OATPs, Pgp, MRPs, etc. Inhibitors of such transporters include but are not limited to: cyclosporine, ritonavir, saquinavir, lopinavir and certain other protease inhibitors, efavirenz, emtricitabine, verapamil, clarithromycin, quinine, quinidine, disopyramide etc.
- Enzyme inducing substances such as rifampicin, carbamazepine, phenytoin, St John´s wort (Hypericum perforatum).
- Warfarin or other anticoagulants in therapeutic dosages.

No concomitant radiotherapy is allowed while on study treatment. However, after surgery radiotherapy should be delivered as per local practices standards.

9. Criteria of evaluation

9.1. Safety evaluation

The following safety parameters will be assessed during treatment: physical examination, medical history, vital signs, 12-lead ECGs, Echocardiogram with LEVF and laboratory assessments. Section 6 provides details on the type and timing of these assessments.

All patients will be evaluable for safety from the time of their first treatment with E7389.

AEs will be graded according to the NCI-CTC version 4.

Please refer to Section 10 for details on general safety assessments / evaluations.

9.2. Efficacy evaluation

The primary endpoints is pathologic complete response to E7389. Patients will be divided in breast responders and breast/axilla responders as follows:

- *Breast Pathological complete responders*: those patients who achieve a pCR of the breast in the surgical specimen.
- *Breast and Axilla Pathological complete responders:* those patients who achieve a pCR of the breast and of the axilla in the surgical specimen.

9.3. Pathological evaluation

pCR_B is defined as the complete absence of invasive cancer in the breast at the time of the definitive surgery, regardless of axillary status or presence of carcinoma *in situ* (CIS). The pCR assessment will be made in local laboratories and no central laboratory confirmation is mandatory. The presence / absence of CIS will be documented.

pCR_{BA} is defined as the complete absence of invasive cancer in the breast and axillary lymph nodes at the time of the definitive surgery.

9.3.1. Definition of Clinical response

Clinical response will be evaluated by physical examination and imaging techniques, and according to the RECIST 1.1 criteria. All eligible patients will be included in the response assessment. All patients who have received at least one treatment and have their disease re-evaluated, either by physical examination or by imaging techniques, will be assigned a response category according to RECIST 1.1 (CR, PR, SD or PD; see definitions below).

For the purpose of clinical response determination, patients who interrupt treatment before of the 8 planned administrations of chemotherapy (for toxicity or other reasons) will be classified according to the last evaluation of the disease prior to surgery or to the beginning of another chemotherapy scheme.

Response criteria are essentially based on the measurable lesion identified at baseline as “target lesion”, and followed until surgery or disease progression.

9.3.2. Measurability of tumor lesions

Definitions

- **Target lesion**: breast primary tumor lesion that can be accurately measured in at least one dimension (longest diameter to be recorded) as ≥ 20 mm by physical examination and/or mammography and breast US or breast MRI and US (where available). All tumor measurements must be recorded in millimeters (or decimal fractions of centimeters) by use of a ruler or caliper.

- **In the case of a multifocal tumor**: if a multifocal tumor is present (defined as the presence of two or more foci of cancer within the same breast quadrant), the largest lesion must be ≥ 20 mm and designated as “target lesion” for all subsequent tumor evaluations. The other focus of tumor will be classified as “Non-target lesions”.

All baseline evaluations should be performed as closely as possible to the beginning of treatment and never more than 2 weeks before its beginning.

Methods of measurements

The same method of assessment and the same technique should be used to characterize the target lesion at baseline and during follow-up. Assessments should be identified on a calendar schedule. While on study, the target lesion recorded at baseline should have the actual measurements recorded on the CRF at each subsequent evaluation, even when very small. If it is the opinion of the radiologist that the lesion has likely disappeared, the measurement should be recorded as 0 mm. If the lesion is believed to be present and is faintly seen but too small to measure, a default value of 5 mm should be assigned.

Timing of measurements

The clinical response will be evaluated as follows:

- At the Screening Visit, immediately before Day 1 of each cycle and at the pre-surgery visit by means of physical examination

  and

- At the Screening Visit, immediately before Cycle 1 Day 1 of eribulin and at the pre-surgery visit by mammography and breast US or MRI and US. The same imaging technique selected at baseline must be consistently used throughout the trial for tumor assessment.

9.3.3. Tumor response evaluation

Tumor response will be classified according to the RECIST 1.1 criteria.

All patients will have their BEST RESPONSE from the start of study treatment until the end of treatment classified, as outlined below.

Complete responses may be claimed only if the criteria for each are met 1 week after initial CR determination.

Definitions

- Complete Response (CR): disappearance of all *target* and *non-target* lesions. Residual lesions thought to be non-malignant should be further investigated before CR can be accepted.
- Partial Response (PR): at least a 30% decrease in the sum of measures of target lesions, taking as reference the baseline sum of diameters. Non target lesions must be non-PD.
- Stable Disease (SD): Neither sufficient shrinkage to qualify for PR nor sufficient increase to qualify for PD taking as reference the smallest sum of diameters on study.
- Progressive Disease (PD): at least a 20% increase in the sum of diameters of measured lesions taking as references the smallest sum of diameters recorded on study (including baseline) AND an absolute increase of ≥ 5 mm. Appearance of new lesions will also constitute PD (including lesions in previously unassessed areas). In exceptional circumstances, unequivocal progression of non-target disease may be accepted as evidence of disease progression, where the overall tumour burden has increased sufficiently to merit

discontinuation of treatment, for example where the tumour burden appears to have increased by at least 73% in volume (which is the increase in volume when all dimensions of a single lesion increase by 20%). Modest increases in the size of one or more non-target lesions are NOT considered unequivocal progression. If the evidence of PD is equivocal (target or non-target), treatment may continue until the next assessment, but on further documentation, the earlier date must be used.

Table 6. Integration of target, non-target and new lesions into response assessment:

**Target Lesions**	**Non-Target Lesions**	**New Lesions**	**Overall Response**
**CR**	CR	No	CR
**CR**	Non-CR/Non-PD	No	PR
**CR**	Not all evaluated	No	PR
**PR**	Non-PD/ not all evaluated	No	PR
**SD**	Non-PD/ not all evaluated	No	SD
**Not all evaluated**	Non-PD	No	NE
**PD**	Any	Any	PD
**Any**	PD	Any	PD
**Any**	Any	Yes	PD
**No Target**	CR	No	CR
**No Target**	Non-CR/non-PD	No	Non-CR/ non-PD
**No Target**	Not all evaluated	No	NE
**No Target**	Unequivocal PD	Any	PD
**No Target**	Any	Yes	PD

10. Patient Safety

10.1. Safety endpoints assessed in this trial

The NCI CTCAE v.4.0 will be used in this study to grade clinical and laboratory adverse events. Laboratory, vital signs and 12-lead ECG abnormalities are to be recorded as adverse events. Safety profile will be based on incidence, severity, chronicity and cumulative nature of treatment emergent AEs (TEAEs). TEAEs are defined as AEs that develop or worsen in grade or become serious during the on-treatment period.

AEs, starting after the informed consent is signed but resolved before the start of AT treatment are not collected in the CRF. Pre-treatment and TEAEs will be summarized with respect to the type, frequency, severity, seriousness, and relatedness as classified by the Medical Dictionary for Regulatory Activities (MedDRA, current version or immediate previous version).

Note: Any clinically relevant abnormal laboratory value or ECG parameter will be immediately rechecked for confirmation before making a decision of permanent discontinuation of AT-E7389 for the concerned patient.

Please also refer to Section 10.5 for details on patient temporary or permanent treatment discontinuation and for patient study discontinuation.

10.2. Adverse Events, Severity and Relationship

10.2.1. Definition of Adverse Event

An **adverse event** (AE) is any untoward medical occurrence in a subject or clinical investigation subject administered an investigational product. An adverse event does not necessarily have a causal relationship with the medicinal products.

All adverse events encountered during the clinical study will be reported on the Case Report Form (CRF). All adverse events, regardless of relationship to study drug or procedures, should be collected beginning from the time the subject signs the study consent. Adverse Events in clinical investigation subjects include any change in the subject's condition. This includes symptoms, physical findings or clinical syndromes.

An abnormal laboratory value may be considered an adverse event if the identified laboratory abnormality leads to any type of intervention whether prescribed in the protocol or not. It is up to the investigator to determine whether an abnormal laboratory value constitutes an adverse event.

Examples of laboratory abnormalities which should be considered as adverse events include those which result in withdrawal of the study treatment, withholding study treatment pending some investigational outcome, reduction of dose of the study treatment or additional concomitant treatment. All laboratory abnormalities considered to constitute an adverse event should be reported on the appropriate page of the CRF. Laboratory abnormalities do not need to be listed as separate adverse events if they are considered to be part of a clinical syndrome that is being reported as an adverse event. It is the responsibility of the investigator to review all laboratory findings in all subjects. Abnormal values should be commented upon as to clinical relevance or importance on the CRF or the laboratory report as appropriate. Medical and scientific judgment should be exercised in deciding whether an isolated laboratory abnormality should be classified as an adverse event.

Every effort must be made by the investigator to categorize each adverse event according to its severity and its relationship to the study treatment.

10.2.2. Assessing Severity of Adverse Events

Adverse events will be graded on the five-point scale according to the NCI Common Terminology Criteria (CTC) version 4.0. Where a CTC grade does not exist for the adverse the detail indicated on the Case Report Form. The definitions are as follows:

Table 7. Severity of adverse event

MILD	Discomfort noticed, but no disruption of normal daily activity.
MODERATE	Discomfort sufficient to reduce or affect normal daily activity.
SEVERE	Incapacitating, with inability to work or to perform normal daily activity.

10.2.3. Assessing Relationship to Study Treatment

Items to be considered when assessing the relationship of an adverse event to the study treatment are:

- Temporal relationship of the onset of the event to the initiation of the study treatment.
- The course of the event, considering especially the effect of discontinuation of study treatment or reintroduction of study treatment, as applicable.

- Whether the event is known to be associated with the study treatment, or with other similar treatments.
- The presence of risk factors in the study subject known to increase the occurrence of the event.
- The presence of non-study treatment related factors which are known to be associated with the occurrence of the event.

10.2.4. Classification of Causality

- **Not Related**: A causal relationship between the study treatment and the adverse event is not a reasonable possibility.
- **Related**: A causal relationship between the study treatment and the adverse event is a reasonable possibility. The investigator must further qualify the degree of certainty as "possible" or "probable".

10.2.5. Pregnancy Reporting

Any pregnancy where the estimated date of conception occurred either prior to the study termination visit, or within 30 days of last study treatment must be reported.

If an adverse outcome of a pregnancy is suspected to be related to study drug exposure, this should be reported regardless of the length of time that has passed since the exposure to study treatment.

An induced abortion or a spontaneous abortion is considered to be a serious adverse event and should be reported in the same timeframe and in the same format as all other serious adverse events.

A subject who becomes pregnant must be withdrawn from the study.

10.3. Serious Adverse Events

10.3.1. Definition of Serious Adverse Events

A serious adverse event (experience) or reaction is any untoward medical occurrence which:

- Results in death.
- Is life-threatening (Note: The term "life-threatening" in the definition of "serious" refers to an event in which the subject was at risk of death at the time of the event; it does not refer to an event which hypothetically might have caused death if it were more severe).
- Requires inpatient hospitalization or prolongation of existing hospitalization.
- Results in persistent or significant disability/incapacity.
- Is a congenital anomaly/birth defect.

Medical and scientific judgment should be exercised in deciding whether expedited reporting is appropriate in other situations, such as important medical events that may not be immediately life-threatening or result in death or hospitalization, but may jeopardize the subject or may require intervention to prevent one of the other outcomes listed in the definition above. These should also usually be considered serious.

The following hospitalizations are not considered to be Serious Adverse Events because there is no "adverse event" (i.e. there is no untoward medical occurrence) associated with the hospitalization:

- Hospitalizations for respite care.
- Planned hospitalizations required by the protocol.
- Hospitalization planned prior to informed consent (where the condition requiring the hospitalization has not changed post study drug administration).

- Hospitalization for administration of study drug or insertion of access for administration of study drug

10.3.2. Reporting of Serious Adverse Events

All Serious Adverse Events, irrespective of relationship to study treatment, must be reported as soon as possible but no later than one business day. The “Clinical Trial Serious Adverse Event Form” (SAE form) must be completed immediately and sent to farmacovigilanza,noprofit@istitutotumori.mi.it by mail. It is very important that the serious adverse event report form be filled out as completely as possible at the time of the initial report. This includes the investigator’s assessment of causality. Any follow-up information received on serious adverse events should be forwarded to farmacovigilanza@istitutotumori.mi.it within one business day of its receipt. If the follow-up information changes the investigator’s assessment of causality, this should also be noted on the follow-up SAE form.

Serious adverse events, regardless of causality assessment, must be collected through the termination visit and for 30 days following study drug discontinuation, whichever is longer.

Any serious adverse event judged by the investigator to be related to the study treatment should be reported to the Sponsor regardless of the length of time that has passed since study completion.

Preliminary SAE reports should be followed as soon as possible by detailed descriptions including copies of hospital case reports, autopsy reports and other documents requested by the Sponsor.

The investigator should notify the Institutional / Ethics Committee of the occurrence of the serious adverse event, in writing, in accordance with local requirements.

10.4. Patient temporary or permanent discontinuation and patient study discontinuation

The study treatment should be continued as per protocol whenever possible. Any study treatment discontinuation should be fully documented in the CRF. In any case, the patient should remain in the study as per protocol whenever possible.

Pregnancy will lead to definitive treatment discontinuation and should be reported as a SAE.

10.4.1.Temporary treatment discontinuation of AT or E7389

Temporary treatment discontinuation may be considered by the Investigator because of suspected AEs. Reinitiating of treatment with the study treatment will be done under close and appropriate clinical/and or laboratory monitoring once the Investigator will have considered according to his/her best medical judgment that the responsibility of the study treatment in the occurrence of the concerned event was unlikely and if the selection criteria for the study are still met (please refer to Sections 5.2 and 5.3).

Dose adjustment will be made according to the grade of toxicity and time of delay. Please refer to Sections 8.2.3. and 8.2.4. for details on dose adjustments.

All temporary treatment discontinuation and respective duration should be recorded by the Investigator in the appropriate pages.

10.4.2 Permanent treatment discontinuation of AT or E7389

Permanent treatment discontinuation is any treatment discontinuation associated with the definitive decision from the Investigator or the patient not to re-expose the patient to AT or E7389 at any time.

10.4.3 List of criteria for definitive treatment discontinuation

The patients may withdraw from treatment if they decide to do so, at any time and irrespective of the reason. Alternatively, treatment will be permanently discontinued per Investigator's decision or per protocol, as detailed below.

All efforts should be made to document the reasons for treatment discontinuation and this should be documented in the CRF as:

1. Progressive Disease by clinical evaluation or as documented by RECIST 1.1 Criteria.
2. Losing clinical benefit because of undue toxicity.
3. The patient withdraws consent.
4. The investigator concludes that further therapy is not in the best interest of the patient.
5. Presence of other medical conditions that prohibit continuation of therapy.
6. Pregnancy.
7. Failure of patient to comply with study procedures that compromise safety, despite repeated efforts of the investigator to contact the patient, with complete documentation of the circumstances.
8. A delay of more than 14 days in starting the next cycle due to toxicities, or the presence of residual toxicities that in the opinion of the investigator prohibit further administration of treatment.
9. Presence of new medical information that warrants the termination of the study.
10. Termination of the study by the Sponsor.

10.4.4 Handling of patients after permanent treatment discontinuation

Patients will be followed up according to the study procedures as specified in this protocol up to the scheduled date of study completion, or up to recovery or stabilization of any AE to be followed-up as specified in this protocol, whichever comes last.

After permanent discontinuation of study treatment, all efforts should be done to ensure that the patients will be assessed using the procedure normally planned in the protocol.

All permanent treatment discontinuation should be recorded by the Investigator in the appropriate pages when considered as confirmed.

10.5 Procedure and consequence for patient withdrawal from study

If withdrawal from the study occurs for any reason, the patient should be assessed using the procedure normally planned for the end-of-study visit.

For patients who fail to return to the site, the Investigator should make the best effort to re-contact the patient (e.g., contacting patient's family or private physician, review available registries or health care database), and to determine his/her health status, including at least his/her vital status. Attempts to contact such patients must be documented in the patient's records (e.g., times and dates of attempted telephone contact, receipt for sending a registered letter).

The statistical analysis plan will specify how these patients lost to follow-up for their primary endpoints will be considered.

Patients who have withdrawn from the study cannot be re-enrolled in the study.

11. Statistical considerations

11.1. Statistical design and simple size

This is phase II, non-randomized, exploratory study of AT followed by E7389 as neoadjuvant therapy in patients with stage I-III ER/PR/HER2 negative breast cancer, that aims evaluate pathologic complete response in both breast and axilla.

A Simon's optimal two-stage design is applied. The sample size is estimated to detect a 40% pCR, with 20% for a minimal hypothesis. With a type I error of 0.05 and a statistical power of 80%, in the first stage, 13 patients have to be enrolled. If 3 pCRs or fewer are observed in this first stage, consideration will be given to stop accrual. If 4 pCRs or more are observed, accrual will continue to obtain 43 treated patients overall. At the end of the trial, if more than 13 pCRs are observed among the 43+5 patients treated with AT→ Eribulin the null hypothesis will be rejected. Accrual is planned to stop as soon as the number of required responses by the statistical design are achieved. Estimating and testing of the response rate will be based on the exact binomial distribution.The analysis will be performed on all the subjects who have provided informed consent and who satisfy all baseline screening inclusion and exclusion criteria, and that will receive at least one cycle of chemotherapy.

Descriptive statistics will be used to summarize patient characteristics, diagnoses, treatment administration and compliance, activity endpoints, safety parameters and eventually cancer biomarkers.

Data will also be displayed graphically, when appropriate.

11.2. Accrual and Duration of Study

The estimated accrual for this study is expected to be 3-4 patients per month considering all centers. A minimum of 18 months is expected to complete accrual.

All of the patients registered in the study will be accounted for. The number of patients who were not evaluable or withdrew before treatment began will be specified. The distribution of follow-up time will be described and the number of patients lost to follow-up will be given.

11.3. Dataset description

- Safety dataset: data from all the patients that have received at least one dose of AT.
- Efficacy dataset: data from all the patients who have received at least one dose of AT and that have been submitted to at least one response evaluation after baseline tumor evaluation.

11.4. Analysis Populations

- Intent-to-Treat (ITT) population: includes all patients that are enrolled in the study. This population will be analyzed for the primary endpoint.
- Evaluable Population (EP): includes all the patients who have initial tumor biopsy available and who undergo definitive surgery, and those who discontinue study treatment due to PD. This population will be analyzed for the primary and secondary endpoints of pCR in the breast and axilla as well as the ORR, best overall response rate (BORR) and breast conservation rate (BCR).

- Safety population (SP): includes the set of patients who received at least one (even incomplete) part of the study treatments. This population will be analyzed for the secondary endpoint of safety.

11.5. Baseline characteristics

A tabulation of subject disposition will be presented, including the number of subjects screened, the number of subjects dosed, a summary of the overall population, and the number and reasons for subject withdrawal.

Demographic and baseline characteristic data summarization will be presented overall. Data to be tabulated include demographic features such as age as well as disease-specific status, medical history, weight and ECOG performance status.

11.6. Efficacy variables

To evaluate the rates of response of the patients treated, the proportion of patients classified as responders and non-responders will be tabulated according to the timing of evaluation. The proportion of responders and non-responders will also be tabulated according to the last evaluation.

The proportion of patients who are evaluated as pCR_B, pCR_{BA}, CR, PR, SD or PD will also be tabulated, as well as the proportion of the patients who can undergo BCS after treatment.

The primary analysis of pCR_B rate with their 95% CI will be calculated based on the ITT and EP populations. Patients not evaluable will be considered as censored data. The primary efficacy analysis of pCR_{BA} rates in the breast and axilla with their 95% confidence interval (CI) will be calculated based on the ITT and EP populations. Patients not evaluable will be considered as non-responders.

11.7. Safety Variables

All safety analyses will be performed on the Safety Population. All adverse events will be graded according to the NCI CTCAE v.4.0.

Overview of AEs summarizing the number (%) of patients with any AE, any grade 3-4 AE, any serious AE, any AE leading to death, any AE leading to dose reduction, any AE leading to dose delay and any AE leading to permanent treatment discontinuation.

Summary of the number (%) of patients with specific AEs of neutropenia, neuropathy, myalgia/arthralgia will be presented.

The number (%) of patients who died by study period (on-study, on-treatment) and the cause of death (disease progression, adverse events, other) will be tabulated.

The clinical laboratory data (including hematology, clinical chemistry, INR) will be graded according to the NCI CTCAE v.4.0 scale, when applicable. When the NCI CTCAE scale is not applicable, the out-of-normal laboratory range value analysis will be performed. Clinical laboratory values will be analyzed after conversion into standard international units.

Appropriate summary statistics will be provided for the analyses of vital signs (i.e. weight, blood pressure, heart rate, and body temperature), ECOG performance status, and ECGs.

12. Ethical and regulatory standards

12.1 Ethical principles

This Clinical Trial will be conducted in accordance with the principles laid down by the 18th World Medical Assembly (Helsinki, 1964) and all applicable amendments laid down by the World Medical Assemblies and the ICH guidelines for Good Clinical Practice (GCP).

This clinical trial will be recorded in the public registry website www.clinicaltrials.gov before the enrollment of the first patient. The registry will contain basic information about the trial sufficient to inform interested patients (and their healthcare practitioners) how to enroll in the trial.

12.2 Laws and regulations

This Clinical Trial will be conducted in compliance with all international guidelines, and national laws and regulations of the country(ies) in which the Clinical Trial is performed, as well as any applicable guidelines.

12.3 Informed consent

The Investigator (according to applicable regulatory requirements), or a person designated by the Investigator, and under the Investigator's responsibility, should fully inform the Patient of all pertinent aspects of the Clinical Trial including the written information giving approval/favorable opinion by the Ethics Committee (IRB/IEC). All participants should be informed to the fullest extent possible about the study, in language and terms they are able to understand.

Prior to a patient’s participation in the Clinical Trial, the written Informed Consent Form should be signed, name filled in and personally dated by the patient or by the patient’s legally acceptable representative, and by the person who conducted the informed consent discussion. A copy of the signed and dated written Informed Consent Form will be provided to the patient.

12.4 Institutional Review Board / Independent Ethics Committee

As required by local regulation, the Investigator must submit this Clinical Trial Protocol to the appropriate Ethics Committee (IRB/IEC), and is required to forward to the respective other party a copy of the written and dated approval/favorable opinion signed by the Chairman with Ethics Committee (IRB/IEC) composition.

The Clinical Trial (study number, Clinical Trial Protocol title and version number), the documents reviewed (Clinical Trial Protocol, Informed Consent Form, Investigator’s Brochure, Investigator’s curriculum vitae [CV], etc.) and the date of the review should be clearly stated on the written (IRB/IEC) approval/favorable opinion.

Study medications will not be released at the study site and the Investigator will not start the study before the written and dated approval/favorable opinion is received by the Investigator and the Sponsor.

During the Clinical Trial, any amendment or modification to the Clinical Trial Protocol should be submitted to the Ethics Committee (IRB/IEC) before implementation, unless the change is necessary to eliminate an immediate hazard to the patients, in which case the IRB/IEC should be informed as soon as possible. It should also be informed of any event likely to affect the safety of patients or the continued conduct of the Clinical Trial, in particular any change in safety. All updates to the Investigator’s Brochure will be sent to the Ethics Committee (IRB/IEC).

A progress report is sent to the Ethics Committee (IRB/IEC) at least annually and a summary of the Clinical Trial’s outcome at the end of the Clinical Trial.

13. Study monitoring

13.1 Responsibilities of the Investigator(s)

The Investigator(s) and delegated Investigator staff undertake(s) to perform the Clinical Trial in accordance with this Clinical Trial Protocol, ICH guidelines for Good Clinical Practice and the applicable regulatory requirements.

The Investigator is required to ensure compliance with all procedures required by the Clinical Trial Protocol The Investigator agrees to provide reliable data and all information requested by the Clinical Trial Protocol (with the help of the e-CRF, Discrepancy Resolution Form [DRF] or other appropriate instrument) in an accurate and legible manner. If any circuit includes transfer of data particular attention should be paid to the confidentiality of the patient's data to be transferred.

The Investigator may appoint such other individuals as he/she may deem appropriate as Sub-Investigators to assist in the conduct of the Clinical Trial in accordance with the Clinical Trial Protocol. All Sub-Investigators shall be appointed and listed in a timely manner. The Sub-Investigators will be supervised by and work under the responsibility of the Investigator. The Investigator will provide them with a copy of the Clinical Trial Protocol and all necessary information.

13.2 Source document requirements

According to the ICH guidelines for Good Clinical Practice, the Informed Consent Form will include a statement by which the patient allows the Ethics Committee (IRB/IEC), and the regulatory authorities to have direct access to original medical records These personnel, bound by professional secrecy, must maintain the confidentiality of all personal identity or personal medical information (according to confidentiality and personal data protection rules).

13.3 Use and completion of Case Report Forms (CRFs) and additional request

It is the responsibility of the Investigator to maintain adequate and accurate CRFs to record all observations and other data pertinent to the clinical investigation. All CRFs should be completed in their entirety in a neat, legible manner to ensure accurate interpretation of data.

14. Administrative rules

14.1 Curriculum Vitae

A current copy of the curriculum vitae describing the experience, qualification and training of each Investigator and Sub-Investigator will be signed and dated prior to the beginning of the Clinical Trial. However, the submission of this Clinical Trial Protocol and other necessary documentation to the Ethics Committee (IRB/IEC) is expressly permitted, the IRB/IEC members having the same obligation of confidentiality.

The Sub-Investigators shall be bound by the same obligation as the Investigator. The Investigator shall inform the Sub-Investigators of the confidential nature of the Clinical Trial.

The Investigator and the Sub-Investigators shall use the information solely for the purposes of the Clinical Trial, to the exclusion of any use for their own or for a third party's account.

Furthermore, the Investigator agrees to adhere to the principles of personal data confidentiality in relation to the patients, Investigator and his/her collaborators involved in the study.

15. Property rights

The Investigator shall not mention any information or the Product in any application for a patent or for any other intellectual property rights.

All the results, data, documents and inventions, which arise directly or indirectly from the Clinical Trial in any form, shall be the immediate and exclusive property of the PI.

The PI may use or exploit all the results at his/her own discretion, without any limitation to its property right (territory, field, continuance). The PI shall be under no obligation to patent, develop, market or otherwise use the results of the Clinical Trial.

As the case may be, the Investigator and/or the Sub-Investigators shall provide all assistance required for obtaining and defending any patent, including signature of legal documents.

16. Data protection

- The patient's personal data, which are included in the PI database shall be treated in compliance with all applicable laws and regulations;
- When archiving or processing personal data pertaining to the Investigator and/or to the patients, the PI shall take all appropriate measures to safeguard and prevent access to this data by any unauthorized third party.
- The Sponsor also collects specific data regarding Investigator as well as personal data from any person involved in the study which may be included in the Sponsor’s databases, shall be treated by both the Sponsor and the Investigator in compliance with all applicable laws and regulations.

17. Insurance compensation

It has taken out a liability insurance policy covering this clinical trial. This insurance policy is in accordance with local laws and requirements. An insurance certificate will be provided to the Ethics committees/IRB or Health Authorities in countries requiring this document.

18. PI audits and inspections by regulatory agencies

For the purpose of ensuring compliance with the Clinical Trial Protocol, Good Clinical Practice and applicable regulatory requirements, the Investigator should permit auditing by or on the behalf of the PI and inspection by regulatory authorities.

The Investigator agrees to allow the auditors/inspectors to have direct access to his/her study records for review, being understood that these personnel is bound by professional secrecy, and as such will not disclose any personal identity or personal medical information.

The Investigator will make every effort to help with the performance of the audits and inspections, giving access to all necessary facilities, data, and documents.

As soon as the Investigator is notified of a planned inspection by the authorities, he will inform the PI and authorize the PI to participate in this inspection.

The confidentiality of the data verified and the protection of the patients should be respected during these inspections.

Any result and information arising from the inspections by the regulatory authorities will be immediately communicated by the Investigator to the PI.

The Investigator shall take appropriate measures required by the PI to take corrective actions for all problems found during the audit or inspections.

19. Premature discontinuation of the study or premature close-out of a site

19.1. Decided by the PI in the following cases:

- If the information on the product leads to doubt as to the benefit/risk ratio;
- If the Investigator has received from the PI all IP, means and information necessary to perform the Clinical Trial and has not included any patient after a reasonable period of time mutually agreed upon;
- In the event of breach by the Investigator of a fundamental obligation under this agreement, including but not limited to breach of the Clinical Trial Protocol, breach of the applicable laws and regulations or breach of the ICH guidelines for Good Clinical Practice;
- If the total number of patients are included earlier than expected;

In any case the Sponsor will notify the Investigator of its decision by written notice.

19.2. Decided by the Investigator:

The Investigator must notify (30 days' prior notice) the PI of his/her decision and give the reason in writing.

In all cases (decided by the PI or by the Investigator), the appropriate Ethics Committee(s) (IRB/IEC) and Health Authorities should be informed according to applicable regulatory requirements.

20. Clinical trial results

The PI will be responsible for preparing a CSR and to provide a summary of study results to the Investigator.

21. Publications and communications

The Investigator undertakes not to make any publication or release pertaining to the Study and/or results of the Study prior to the PI’s written consent, being understood that the PI will not unreasonably withhold its approval.

As the Study is being conducted at multiple sites, the PI agrees that, consistent with scientific standards, first presentation or publication of the results of the Study shall be made only as part of a publication of the results obtained by all sites performing the Protocol. However, if no multicenter publication has occurred within twelve (12) months of the completion of this Study at all sites, the Investigator shall have the right to publish or present independently the results of this Study to the review procedure set forth herein. The Investigator shall provide the PI with a copy of any such presentation or publication derived from the Study for review and comment at least thirty (30) days in advance of any presentation or submission for publication. In addition, if requested by the PI,

any presentation or submission for publication shall be delayed for a limited time, not to exceed ninety (90) days, to allow for filing of a patent application or such other measures as the PI deems appropriate to establish and preserve its proprietary rights.

The Investigator shall not use the name(s) of the PI and/or his/her employees in advertising or promotional material or publication without the prior written consent of the PI. The PI shall not use the name(s) of the Investigator and/or the collaborators in advertising or promotional material or publication without having received his/her and/or their prior written consent(s).

The PI has the right at any time to publish the results of the study.

22. Clinical trial protocol amendments

All appendices attached hereto and referred to herein are made part of this Clinical Trial Protocol.

The Investigator should not implement any deviation from, or changes of the Clinical Trial Protocol without agreement by the PI and prior review and documented approval/favorable opinion from the IRB/IEC of an amendment, except where necessary to eliminate an immediate hazard(s) to Clinical Trial Patients, or when the change(s) involves only logistical or administrative aspects of the trial. Any change agreed upon will be recorded in writing, the written amendment will be signed by the Investigator and by the PI and the signed amendment will be filed with this Clinical Trial Protocol.

Any amendment to the Clinical Trial Protocol requires written approval/favorable opinion by the Ethics Committee (IRB/IEC) prior to its implementation, unless there are overriding safety reasons.

In some instances, an amendment may require a change to the Informed Consent Form. The Investigator must receive an IRB/IEC approval/favorable opinion concerning the revised Informed Consent Form prior to implementation of the change and patient signature should be re-collected if necessary.

Appendix A: References

1. Heys SD, Hutcheon AW, Sarkar TK, et al. Neoadjuvant Docetaxel in Breast Cancer: 3-Year Survival Results from the Aberdeen Trial. Clin Breast Cancer 2002; 3(S2):S69-S74

2. Von Minckwitz G, Kümmel S, Vogel P, et al. Neoadjuvant Vinorelbine–Capecitabine Versus Docetaxel–Doxorubicin–Cyclophosphamide in Early Nonresponsive Breast Cancer: Phase III Randomized GeparTrio Trial. J Natl Cancer Inst 2008; 100:542-551

3. Gianni L, Baselga J, Eiermann W, et al. Phase III Trial Evaluating the Addition of Paclitaxel to Doxorubicin Followed by Cyclophosphamide, Methotrexate, and Fluorouracil, As Adjuvant or Primary Systemic Therapy: European Cooperative Trial in Operable Breast Cancer. J Clin Oncol 2009; 27(15):2474-2481

4. Perou CM, Sorlie T, Eisen MB et al. Molecular portraits of human breast tumours. Nature 2000; 406(6797):747-752

5. Sorlie T, Perou CM, Tibshirani R et al. Gene expression patterns of breast carcinomas distinguish tumor subclasses with clinical implications. Proc Natl Acad Sci U S A 2001; 98(19):10869-10874

6. Dent R, Trudeau M, Pritchard KI, et al. Triple-Negative Breast Cancer: Clinical Features and Patterns of Recurrence. Clin Cancer Res 2007;13:4429-4434

7. Carey LA, Dees EC, Sawyer L, et al. The Triple Negative Paradox: Primary Tumor Chemosensitivity of Breast Cancer Subtypes. Clin Cancer Res 2007;13:2329-2334

8. Liedtke C, Mazouni C, Hess KR, et al. Response to neoadjuvant therapy and long-term survival in patients with triple-negative breast cancer. J Clin Oncol 2008; 26(8):1275-81

9. Bear HD. Indications for neoadjuvant chemotherapy for breast cancer. Semin Oncol 1998; 25:3–12

10. Hortobagyi GN, Blumenshein G, Spanos W. Multimodal treatment of locoregionally advanced breast cancer. Cancer 1983; 51: 763

11. Hortobagyi GN, Ames IC, Buzdar AU. Management of stage III primary breast cancer with primary chemotherapy, surgery and radiation therapy. Cancer 1988; 62: 2507

12. Hortobagyi GN. Comprehensive management of locally advanced breast cancer. Cancer 1990; 66:1387–1391

13. Fisher B, Bryant J, Wolmark N, et al. Effect of preoperative chemotherapy on the outcome of women with operable breast cancer . J Clin Oncol 1998; 16:2672–2685

14. Wolmark N, Wang J, Mamounas E, et al. Preoperative chemotherapy in patients with operable breast cancer: nine-year results from National Surgical Adjuvant Breast and Bowel Project B-18. J Natl Cancer Inst Monogr 2001; (30):96-102

15. Bear HD , Anderson S , Brown A , et al. The effect on tumor response of adding sequential preoperative docetaxel to preoperative doxorubicin and cyclophosphamide: preliminary results from National Surgical Adjuvant Breast and Bowel Project Protocol B-27. J Clin Oncol 2003; 21:4165–4174

16. Van der Hage JA, van de Velde CJH, Julien JP, et al. Preoperative Chemotherapy in Primary Operable Breast Cancer: Results From the European Organization for Research and Treatment of Cancer Trial 10902. J Clin Oncol 2001; 19: 4224-4237

17. Kuerer HM, Newman LA, Smith TL, et al. Clinical course of breast cancer patients with complete pathologic primary tumor and axillary lymph node response to doxorubicin-based neoadjuvant chemotherapy. J Clin Oncol 1999; 17(2): 460-9

18. Zhang F, Yang Y, Smith T, et al. Correlation between HER-2 expression and response to neoadjuvant chemotherapy with 5-fluorouracil, doxorubicin, and cyclophosphamide in patients with breast carcinoma. Cancer 2003; 97: 1758–1765

19. Therasse F, Mauriac L, Welnicka-Jaskiewicz M, et al. Final Results of a Randomized Phase III Trial Comparing Cyclophosphamide, Epirubicin, and Fluorouracil With a DoseIntensified Epirubicin and Cyclophosphamide + Filgrastim as Neoadjuvant Treatment in Locally Advanced Breast Cancer: An EORTC-NCIC-SAKK Multicenter Study. J Clin Oncol 2003; 21: 843-850

20. Holmes FA, Walters RS, Theriault RL, et al. Phase II Trial of Taxol, an Active Drug in the Treatment of Metastatic Breast Cancer. J Natl Cancer Inst 1991; 83(24): 1797-1805

21. Gianni L, Munzone E, Capri G, et al. Paclitaxel in metastatic breast cancer: a trial of two doses by a 3-hour infusion in patients with disease recurrence after prior therapy with anthracyclines. J Natl Cancer Inst 1995; 87(15): 1169-75

22. Ravdin PM, Burris HA 3rd, Cook G, et al. Phase II trial of docetaxel in advanced anthracycline-resistant or anthracenedione-resistant breast cancer. J Clin Oncol 1995; 13(12): 2879-85.

23. Valero V, Jones SE, Von Hoff DD, et al. Phase II trial of docetaxel: a new, highly effective antineoplastic agent in the management of patients with anthracycline-resistant metastatic breast cancer. J Clin Oncol 1995; 13: 2886-2894

24. Rastogi P, Anderson SJ, Bear HD, et al. Preoperative Chemotherapy: Updates of National Surgical Adjuvant Breast and Bowel Project Protocols B-18 and B-27. J Clin Oncol 2008; 26: 778-785

25. Mazouni C, Kau SW, Frye D, et al. Inclusion of taxanes, particularly weekly paclitaxel, in preoperative chemotherapy improves pathologic complete response rate in estrogen receptor-positive breast cancers. Ann Oncol 2007; 18: 874–880

26. Smith IC, Heys SD, Hutcheon AW, et al. Neoadjuvant Chemotherapy in Breast Cancer: Significantly Enhanced Response With Docetaxel. J Clin Oncol 2002; 20:1456-1466

27. Kaufmann M, Hortobagyi GN, Goldhirsch A, et al. Recommendations from an international expert panel on the use of neoadjuvant (primary) systemic treatment of operable breast cancer: an update. J Clin Oncol 2006; 24(19): 3221

28. Bear HD, Anderson S, Smith RE, et al. Sequential preoperative or postoperative docetaxel added to preoperative doxorubicin plus cyclophosphamide for operable breast cancer: National Surgical Adjuvant Breast and Bowel Project Protocol B-27. J Clin Oncol 2006; 24: 2019–2027

29. Von Minckwitz G, Raab G, Caputo A, et al. Doxorubicin With Cyclophosphamide Followed by Docetaxel Every 21 Days Compared With Doxorubicin and Docetaxel Every 14 Days As Preoperative Treatment in Operable Breast Cancer: The GEPARDUO Study of the German Breast Group, J Clin Oncol 2005 ; 23: 2676-2685

30. Von Minckwitz G, Rezai M, Loibl S, et al. Capecitabine in Addition to Anthracycline- and TaxaneBased Neoadjuvant Treatment in Patients With Primary Breast Cancer: Phase III GeparQuattro Study. J Clin Oncol 2010; 28(12): 2024-31

31. Chang HR, Glaspy J, Allison MA, et al. Differential response of triple-negative breast cancer to a docetaxel and carboplatin-based neoadjuvant treatment. Cancer 2010; 116(18): 4227-37

32. Torrisi R, Balduzzi A, Ghisini R, et al. Tailored preoperative treatment of locally advanced triple negative (hormone receptor negative and HER2 negative) breast cancer with epirubicin, cisplatin, and infusional fluorouracil followed by weekly paclitaxel. Cancer Chemother Pharmacol 2008

33. Silver DP, Richardson AL, Eklund AC, et al. Efficacy of neoadjuvant Cisplatin in triple-negative breast cancer. J Clin Oncol 2010; 28(7): 1145-53

34. Byrski T, Gronwald J, Huzarski T, et al. Pathologic complete response rates in young women with BRCA1-positive breast cancers after neoadjuvant chemotherapy. J Clin Oncol 2010; 28(3): 375-9

35. Von Minckwitz G, Eidtmann H, Rezai M, et al. Neoadjuvant Chemotherapy and Bevacizumab for HER2-Negative Breast Cancer. N Engl J Med 2012; 366: 299-309

36. Bear HD, Tang G, Rastogi P, et al. Bevacizumab Added to Neoadjuvant Chemotherapy for Breast Cancer. N Engl J Med 2012; 366: 310-320

37. Buzzoni R, Bonadonna G, Valagussa P, et al. Adjuvant chemotherapy with doxorubicin plus cyclophosphamide, methotrexate, and fluorouracil in the treatment of resectable breast cancer with more than three positive axillary nodes. J Clin Onc 1991; 9(12): 2134-40.

38. Poole CJ, Earl HM, Hiller L, et al. Epirubicin and cyclophosphamide, methotrexate, and fluorouracil as adjuvant therapy for early breast cancer. N Engl J Med 2006; 355(18):1851-62.

39. Bonadonna G, Zambetti M, Valagussa P. Sequential or Alternating Doxorubicin and CMF Regimens in Breast Cancer With More Than Three Positive Nodes Ten-Year Results JAMA 1995; 273(7): 542-547

40. Bonadonna G,Valagussa P. Lessons From the Initial Adjuvant Cyclophosphamide, Methotrexate, and Fluorouracil Studies in Operable Breast Cancer. J Clin Oncol 2008; 26: 342-344

41. Tancini C, Bonadonna G, Valagussa P. Adjuvant CMF in breast cancer: comparative 5-year results of 12 versus 6 cycles. J Clin Oncol 1983; 1: 2-10

42. Fisher B, Brown AM, Dimitrov NV, et al. Two months of doxorubicin-cyclophosphamide with and without interval reinduction therapy compared with 6 months of cyclophosphamide, methotrexate, and fluorouracil in positive-node breast cancer patients with tamoxifen-nonresponsive tumors: results from the National Surgical Adjuvant Breast and Bowel Project B-15. J Clin Oncol 1990; 8(9): 1483-96

43. Gianni L, Baselga J, Eiermann W, et al. Feasibility and tolerability of sequential doxorubicin/paclitaxel followed by cyclophosphamide, methotrexate, and fluorouracil and its effects on tumor response as preoperative therapy. Clin Cancer Res 2005; 11(24 Pt 1): 8715-21

44. Gianni L, Zambetti M, Clark K et al. Gene expression profiles in paraffin-embedded core biopsy tissue predict response to chemotherapy in women with locally advanced breast cancer. J Clin Oncol 2005; 23(29): 7265-7277

45. De Laurentiis M, Cancello G, D'Agostino D, et al. Taxane-based combinations as adjuvant chemotherapy of early breast cancer: a meta-analysis of randomized trials. J Clin Oncol 2008; 26(1): 44-53

46. Francis P, Crown J, Di Leo A, et al. Adjuvant chemotherapy with sequential or concurrent anthracycline and docetaxel: Breast International Group 02-98 randomized trial. J Natl Cancer Inst 2008; 100(2): 121-33.

47. Towle MJ, Salvato KA, Budrow J, et al. In vitro and in vivo anticancer activities of synthetic macrocyclic ketone analogues of halichondrin B. Cancer Res 2001; 61(3): 1013-1021

48. Okouneva T, Azarenko O, Wilson L, et al. Inhibition of centromere dynamics by eribulin (E7389) during mitotic metaphase. Mol Cancer Ther 2008; 7(7): 2003-2011

49. Jordan MA, Kamath K, Manna T, et al. The primary antimitotic mechanism of action of the synthetic halichondrin E7389 is suppression of microtubule growth. Mol Cancer Ther 2005; 4(7): 1086-1095

50. Dabydeen DA, Burnett JC, Bai R, et al. Comparison of the activities of the truncated halichondrin B analog NSC 707389 (E7389) with those of the parent compound and a proposed binding site on tubulin. Mol Pharmacol 2006; 70(6): 1866-1875

51. Kuznetsov G, Towle MJ, Cheng H, et al. Induction of morphological and biochemical apoptosis following prolonged mitotic blockage by halichondrin B macrocyclic ketone analog E7389. Cancer Res 2004; 64(16): 5760-5766

52. Goel S, Mita AC, Mita M, et al. A phase I study of eribulin mesylate (E7389), a mechanistically novel inhibitor of microtubule dynamics, in patients with advanced solid malignancies. Clin Cancer Res 2009; 15(12): 4207-4212

53. Synold TW, Morgan RJ, Newman EM. A phase I pharmacokinetic, target validation study of the anti-tubulin agent E7389: a California Cancer consortium trial. J Clin Oncol 23, 200s. 2005. Ref Type: Abstract

54. Tan AR, Rubin EH, Walton DC, et al. Phase I study of eribulin mesylate administered once every 21 days in patients with advanced solid tumors. Clin Cancer Res 2009;15(12):4213-4219

55. Minami H, Mukohara T, Nagai S, et al.A phase I study of eribulin mesylate (E7389) in patients with refractory cancers. Eur J Cancer Suppl 6, 140. 2008. Ref Type: Abstract

56. Vahdat LT, Pruitt B, Fabian CJ, et al. Phase II study of eribulin mesylate, a halichondrin B analog, in patients with metastatic breast cancer previously treated with an anthracycline and a taxane. J Clin Oncol 2009; 27(18): 2954-2961

57. Cortes J, Vahdat L, Blum JL, et al. Phase II study of the halichondrin B analog eribulin mesylate in patients with locally advanced or metastatic breast cancer previously treated with an anthracycline, a taxane, and capecitabine. J Clin Oncol 2010; 28(25): 3922-3928

58. Iwata H, Aogi K, Masuda N, et al. Efficacy an safety of eribulin in Japanese patients (pts) with advanced breast cancer. J Clin Oncol 28, 1081. 2010. Ref Type: Abstract

59. Cortes J, O'Shaughnessy J, Loesch D, et al. Eribulin monotherapy versus treatment of physician's choice in patients with metastatic breast cancer (EMBRACE): a phase 3 open-label randomised study. Lancet 2011; 377(9769): 914-923

60. Twelves C, Cortes J, Vahdat LT, et al. Phase III trials of eribulin mesylate (E7389) in extensively pretreated patients with locally recurrent or metastatic breast cancer. Clin Breast Cancer 2010; 10(2):160-163

61. Gupta A, Mould DR, Reyderman L, et al. Population pharmacokinetic-pharmacodynamic (PK/PD) analysis of safety and efficacy of eribulin in metastatic or locally advanced breast cacer patients. Ann.Oncol 21[Suppl8], 111. 2010. Ref Type: Abstract

62. Loesch DM, Greco F, O’Shaughnessy J, et al. A randomized, multicenter phase III trial comparing doxorubicin + cyclophosphamide followed by paclitaxel or doxorubicin + paclitaxel followed by weekly paclitaxel as adjuvant therapy for high-risk breast cancer. ASCO Annual Meeting Proceedings (Post-Meeting Edition). Vol 25, No 18S (June 20 Supplement), 2007: 517

63. Pean E, Klaar S, Berglund EG, et al. The European Medicines Agency Review of Eribulin for the Treatment of Patients with Locally Advanced or Metastatic Breast Cancer: Summary of the Scientific Assessment of the Committee for Medicinal Products for Human Use Clin Cancer Res 2012; 10: 1158-1078

64. Cochet A, Generali D, Fox SB, et al. Positron Emission Tomography and Neoadjuvant Therapy of Breast Cancer. J Natl Cancer Inst 2011; (43): 111 - 115

Appendix A: ECOG performance status

**Grade**	**ECOG**
**0**	Fully active, able to carry on all pre-disease performance without restriction
**1**	Restricted in physically strenuous activity but ambulatory and able to carry out work of a light or sedentary nature, e.g., light house work, office work
**2**	Ambulatory and capable of all self care but unable to carry out any work activities.<br>Up and about more than 50% of waking hours
**3**	Capable of only limited self care, confined to bed or chair more than 50% of waking hours
**4**	Completely disabled.<br>Cannot carry on any self care.<br>Totally confined to bed or chair
**5**	Dead
